# *OsARD4* encoding an *acireductone dioxygenase* improves root architecture in rice by promoting development of secondary roots

**DOI:** 10.1038/s41598-018-34053-y

**Published:** 2018-10-24

**Authors:** Valarmathi Ramanathan, Hifzur Rahman, Saravanan Subramanian, Jagadeeshselvam Nallathambi, Ashokkumar Kaliyaperumal, Sudha Manickam, Chandrababu Ranganathan, Raveendran Muthurajan

**Affiliations:** Department of Plant Biotechnology, Centre for Plant Molecular Biology and Biotechnology, Tamil Nadu Agricultural University, 641 003 Coimbatore, India

## Abstract

This study was aimed at unravelling the molecular basis of root growth behavior in a drought-tolerant upland rice genotype, Nootripathu. Root tips of Nootripathu were found to possess shorter root caps and a greater number of dividing cells, favoring faster elongation compared to shallow-rooted IR20. Width and length of cortical cells in the roots of rapidly growing Nootripathu were found to be two to three times higher than IR20. Evaluation of shallow-rooted IR20, deep-rooted Nootripathu and their Recombinant Inbred Lines (RILs) for root characteristics revealed the presence of genetic variation for root traits among RILs. 2D-PAGE analysis of proteins in roots of IR20, Nootripathu and bulks of extreme RILs differing in root traits resulted in the identification of proteins co-segregating with root growth behavior and co-localized with QTLs for root traits. A putative candidate gene, *OsARD4*, encoding an “*acireductone dioxygenase*” was validated for its role in modulating the root growth pattern through genetic transformation. Transgenic ASD16 rice plants engineered for the overexpression of *OsARD4* exhibited root growth characteristics similar to those of Nootripathu, including faster radical emergence, more rapid elongation of primary roots, early initiation of crown/lateral roots, and higher root biomass than the non-transgenic plants.

## Introduction

Rice (*Oryza sativa* L.) production has to be raised to approximately 160 million tons by 2050 from the current level of 100 million tons to ensure food security for the increasing global population. To increase the production rate, it is necessary to overcome challenges, *viz*., the yield plateau, diminishing resources, and changing climate^1^. Development of resilient rice genotypes adapted to drought-prone marginal environments that occupy >40% of the area under rice cultivation is becoming essential. Conventional breeding approaches have achieved only limited success because of the complexity of the physiological, genetic and molecular mechanisms involved and the limitations of reliable screening procedures.

Drought avoidance is vital in maintaining productivity under water-limited environments. It is achieved by maintaining high internal water status through increased absorption of water facilitated by an extensive root system and reduced water loss through the canopy^2^. Apart from water uptake, roots also play a major role in the acquisition of nutrients, providing anchorage (non-lodging), synthesis of growth regulators, and production of organic acids to solubilize minerals in the soil^3^. Hence, root growth-related traits are considered important for sustaining rice productivity under drought and nutrient deficient stresses^4^. Traditional rice genotypes possessing deep roots exhibit better drought avoidance^5,6^. Several breeding programs have been initiated to improve the root system architecture in rice but attained only limited success because of the inherent difficulties involved in phenotyping and limited knowledge of the molecular genetic basis of root growth behavior^7^.

Advancements in genomics have accelerated the identification of QTLs/genes controlling root architecture-related traits in rice^2,8,9^. Although more than 700 QTLs controlling various root traits, namely, root number, root length and root thickness, have been mapped^10,11^, only a few candidate genes have been cloned^2,12,13^. Until recently, candidate gene discovery in rice roots through whole genome gene expression profiling remained largely unexplored because of the lack of suitable genetic materials and inherent difficulties in sampling.

Our earlier studies revealed that some of the rice landraces, namely, Nootripathu, Norungan and Kallurundaikar, possess desirable root characteristics for avoiding drought^14^ and were utilized in the mapping of consistent QTLs controlling plant performance under drought stress^15^. This study was aimed at identifying putative candidate genes regulating root growth behavior in the landrace Nootripathu using recent advancements in genomics. 2D-PAGE profiling of proteins was carried out in roots of contrasting rice genotypes, such as IR20, Nootripathu, and bulks of extreme RILs exhibiting contrasting root growth characteristics to identify proteins co-segregating with root growth behavior. Overexpression of a putative candidate gene encoding for “*acireductone dioxygenase* (*OsARD4*),” involved in ethylene and polyamine biosynthesis in a shallow-rooted rice genotype, ASD16, revealed its role in promoting crown/lateral root development and, thereby, modulating rooting patterns.

## Results

### IR20 and Nootripathu differ in their root growth characteristics

**Figure 1:**
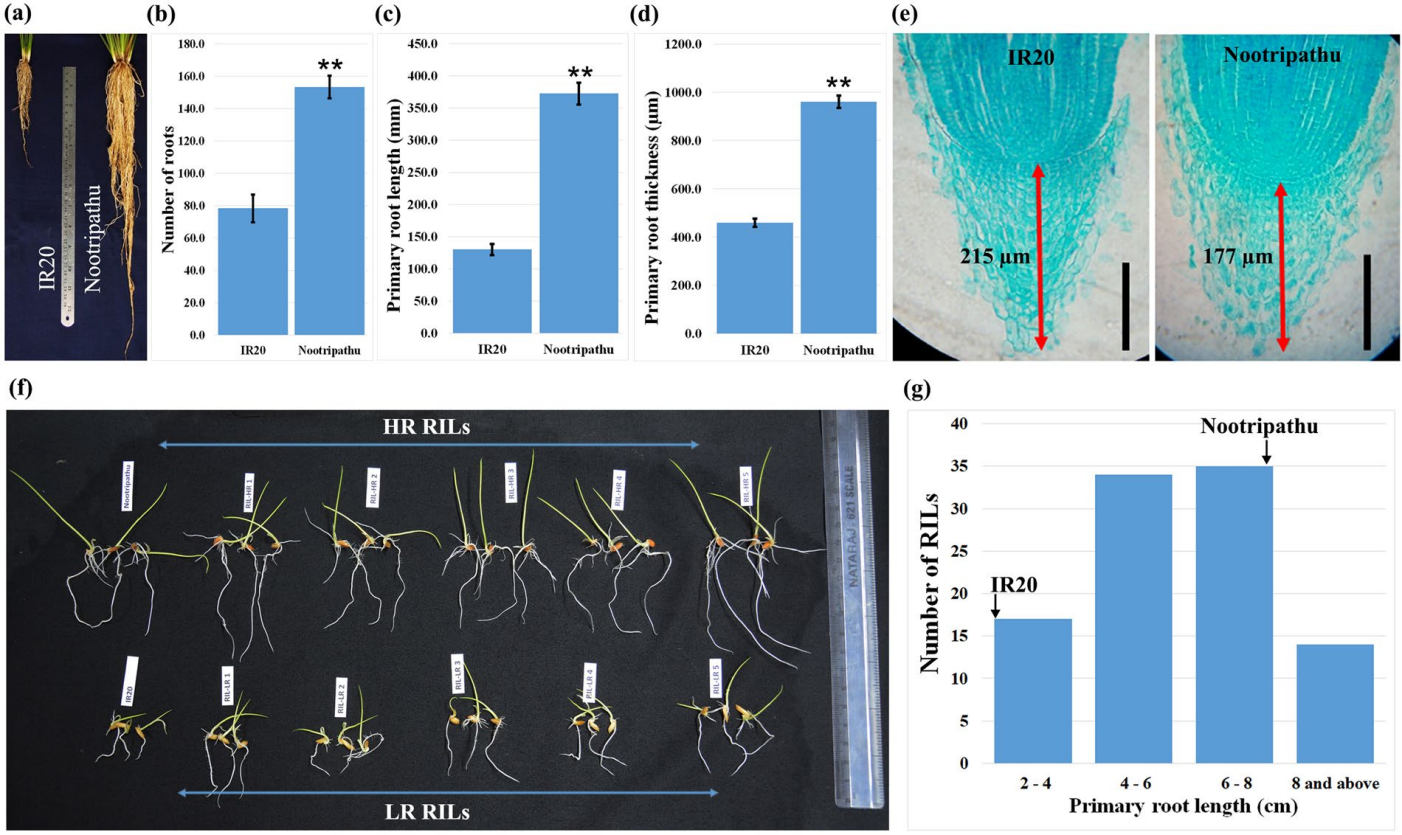
Root growth characteristics of IR20 and Nootripathu. **(a**) Root morphology in 45-day old plants of IR20 and Nootripathu under well-watered conditions. **(b–d**) Mean root number, primary root length and mean root thickness in 45-day old plants of IR20 and Nootripathu. Each data point is the mean of 3 replicates. **(e)** Microscopic examination of longitudinal sections of root tips in 5-day old seedlings of IR20 and Nootripathu. Scale bar = 100 µm. **(f)** Genetic variation for root growth behavior in 8-day old seedlings of IR20, Nootripathu and their selected RILs (HR,high root; LR, low root). **(g**) Frequency distribution of primary root length in 8-day old seedlings of IR20, Nootripathu and their RILs. Each data point is the mean of 5 independent measurements. *Indicates significance at P < 0.05, and **Indicates significance at P < 0.01 (one-way ANOVA compared with IR20).

Landrace Nootripathu has a higher root number and deeper and thicker roots compared to IR20 (Fig. 1a–d). Nootripathu roots have shorter root caps (177.5 µm) compared to IR20 (215 µm) and are found to contain more cells possessing increased length and width in divisional and elongation zones (Fig. 1e; Supplementary Table 1). The final lengths and widths of matured cortical cells were also found to be significantly higher in roots of Nootripathu than in those of IR20 (Supplementary Fig. 1). Rice genotypes IR20 and Nootripathu differed significantly in their root elongation behavior during germination, and Nootripathu exhibited significantly longer primary roots (7.38 cm) than IR20 (2.74 cm) (Fig. 1f,g). RILs derived between them exhibited significant genetic variation for primary root length (2.38 cm to 8.83 cm) on 8 DAS (Fig. 1f,g). Nootripathu showed higher root penetration ability (RPA) (22%) than IR20 (4.6%) through the wax petrolatum layer (WPL), simulating hardpans in soil (Supplementary Fig. 2). RILs exhibited considerable genetic variation in the total number of roots, number of penetrated roots, and root penetration ability (0–46.6%), showing their suitability for mapping and functional genomics studies. RILs exhibiting root characteristics similar to low and shallow-rooted rice genotype IR20 (RIL #354, 264 and 314) were constituted as low root bulk, and RILs with root characteristics similar to upland rice genotype Nootripathu (RIL #61, 361 and 194) were constituted as high root bulk (Supplementary Fig. 2).

### Protein profiling in roots of IR20, Nootripathu and extreme bulks of RILs identified novel proteins co-segregating with root growth behavior

**Figure 2:**
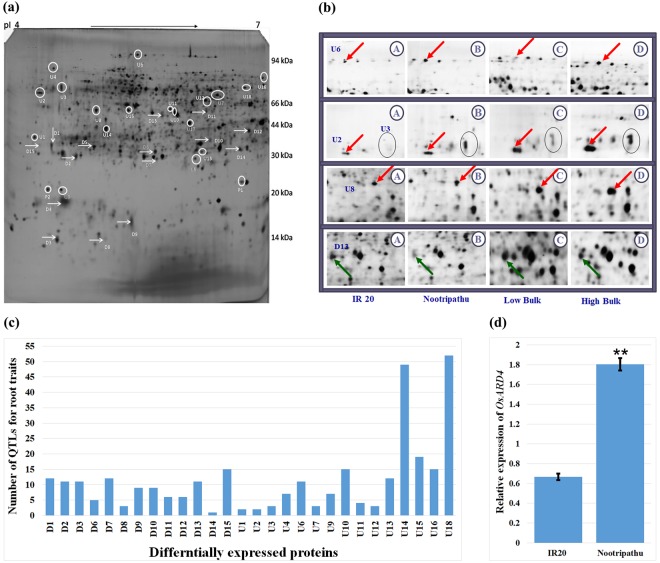
(**a)** Two-dimensional gel electrophoretic pattern of proteins extracted from root tissues (tips) in rice *cv*. Nootripathu. Arrows and circles indicate proteins exhibiting a differential expression pattern between IR20 and Nootripathu. (**b)** Expression pattern of selected proteins in IR20, Nootripathu and contrasting bulks (red arrows indicate proteins showing upregulation in roots of Nootripathu as well as high root bulk, and green arrows indicate proteins showing downregulation in roots of Nootripathu and high root bulk). (**c)** Graph showing co-localization of differentially expressed proteins within the boundary of QTLs for root growth-related traits. (**d)** qRT-PCR analysis of *OsARD4* transcripts in root tissues of IR20 and Nootripathu. Error bars show standard errors between three biological replicates. **Indicates significance at P < 0.01 (one-way ANOVA compared with IR20).

Protein profiling in roots of IR20 and Nootripathu identified differentially expressed (DE) proteins between them, and a comparison of the abundance of corresponding proteins in the roots of extreme bulks of RILs resulted in the identification of proteins co-segregating with root growth behavior (Fig. 2a,b). Of the 33 proteins showing differences in their abundance between parents and between bulks of RILs, 8 proteins showed downregulation and 10 proteins showed upregulation in roots of Nootripathu compared to IR20 (Table 1). Among the 10 proteins upregulated in the roots of Nootripathu compared to IR20, 6 proteins showed upregulation in the roots of high root bulks over low root bulks. Of the 8 proteins showing downregulation in roots of Nootripathu compared to IR20, 6 proteins were found to be downregulated in the roots of high root bulks over low root bulks (Table 1).

**Table 1:** Abundance ratio and putative function of differentially expressed proteins identified by MALDI-TOF MS.

Spot ID	Protein Name	Locus ID	MS	Theoretical		Experimental		MP/PS	SC%	Abundance ratio	
				pI	Mr	pI	Mr			Nootripathu/IR20	High Bulk/Low Bulk
D1	Putative ADP-ribosylation factor	LOC_Os03g27450.1	60	5.42	22.0	4.47	42	6/23	38	**0.43** ± **0.07**	0.43 ± 0.01
D2	Cell wall adhesion	LOC_Os11g11730.1	78	4.45	48.5	4.84	35	11/21	38	0.49 ± 0.09	0.53 ± 0.13
D3	Retrotransposon protein	LOC_Os01g54700.1	58	7.8	20.5	4.55	12	12/30	43	**0.33** ± **0.03**	0.49 ± 0.02
D6	Auxin response factor	LOC_Os06g47150.4	73	8.67	52.3	5.81	39	8/23	17	1.00 ± 0.13	0.84 ± 0.41
D7	Expressed protein	LOC_Os01g56790.1	63	5.95	16.4	5.82	36	7/24	52	**0.37** ± **0.02**	0.37 ± 0.23
D8	Oxidoreductase, aldo/ketoreductase family protein	LOC_Os02g03100.2	61	9.16	28.5	5.19	12	7/36	34	**0.33** ± **0.12**	**0.24** ± **0.03**
D9	Maturase K	LOC_Os04g16734.1	58	9.8	33.8	5.52	17	22/35	49	0.53 ± 0.10	0.64 ± 0.12
D10	Chloroplast 30S ribosomal protein S3	LOC_Os04g16824.1	76	10.0	25.3	6.45	42	10/13	43	0.47 ± 0.11	**0.34** ± **0.07**
D11	MAR-binding filament-like protein 1	LOC_Os01g08510.1	72	5.83	80.2	6.40	53	21/36	25	0.54 ± 0.07	0.47 ± 0.08
D12	Proteophosphoglycan ppg4	LOC_Os05g10830.2	71	12.2	25.1	6.89	47	7/36	36	**0.52** ± **0.02**	0.53 ± 0.05
D13	Disease resistance protein RPM1	LOC_Os11g12040.1	67	6.3	77.9	5.90	52	30/33	39	**0.53** ± **0.03**	0.39 ± 0.05
D14	PPR repeat domain containing protein	LOC_Os10g36190.1	62	7.47	55.1	6.78	38	15/25	19	0.44 ± 0.09	0.63 ± 0.22
D15	Ras-related protein	LOC_Os02g37420.1	61	6.9	23.4	4.40	43	12/23	71	0.37 ± 0.05	**0.47** ± **0.02**
U1	Hexokinase	LOC_Os07g09890.1	47	6.49	55.0	4.40	47	5/13	11	1.88 ± 0.05	2.13 ± 0.25
U2	Protein kinase family protein	LOC_Os08g02050.1	68	8.37	58.6	4.41	59	14/14	13	2.40 ± 0.33	**2.43** ± **0.25**
U3	1,2-dihydroxy-3-keto-5-methylthiopentene dioxygenase	LOC_Os10g28360.1	60	5.07	22.1	4.63	60	6/30	33	**4.53** ± **0.02**	2.15 ± 0.04
U4	Peroxiredoxin	LOC_Os07g44430.1	48	5.97	22.2	4.50	85	4/20	31	17.19 ± 0.2	3.38 ± 0.18
U6	Annexin	LOC_Os07g46550.1	68	6.2	20.1	5.60	90	9/18	36	1.77 ± 0.21	**2.01** ± **0.08**
U7	Leucine rich repeat containing protein	LOC_Os08g40090.1	41	6.83	62.3	6.52	57	21/22	24	2.56 ± 0.02	**2.08** ± **0.10**
U9	Expressed protein	LOC_Os04g24610.1	70	4.88	56.3	4.60	24	8/29	25	**2.05** ± **0.20**	1.81 ± 0.55
U10	Pathogenesis-related Bet v I family protein	LOC_Os03g18850.1	63	6.04	12.9	6.05	53	5/20	34	**2.06** ± **0.09**	**2.03** ± **0.01**
U11	CGMC_MAPKCMGC_2_ERK.13 - CGMC includes CDA, MAPK, GSK3, and CLKC kinases	LOC_Os08g06060.1	55	8.05	42.9	6.00	54	8/20	32	1.91 ± 0.15	**2.71** ± **0.33**
U12	Glutaredoxin	LOC_Os05g28530.1	60	9.28	26.5	6.40	56	6/18	25	**2.03** ± **0.01**	1.66 ± 0.13
U13	RNA recognition motif	LOC_Os04g50790.1	53	5.85	33.5	6.36	38	10/28	31	1.73 ± 0.02	1.93 ± 0.64
U14	Expressed protein	LOC_Os12g06560.1	76	6.39	117.9	5.22	48	17/22	15	2.54 ± 0.11	1.79 ± 0.06
U15	Leaf senescence related protein	LOC_Os01g68630.1	50	5.69	67.3	5.49	54	7/25	14	2.13 ± 0.47	**1.54** ± **0.06**
U16	Ras-related protein	LOC_Os07g31370.1	75	8.58	21.1	6.95	64	8.42	44	9.73 ± 1.19	**2.36** ± **0.05**
U17	NADPH reductase	LOC_Os09g38620.1	65	5.38	79	6.22	50	11/40	17	1.59 ± 0.05	2.35 ± 0.89
U18	NBS-LRR disease resistance protein	LOC_Os11g45180.1	71	6.26	118	6.84	59	11/32	13	1.59 ± 0.35	1.99 ± 0.46

Homologous proteins were identified by MASCOT analysis along with their experimental and theoretical pI and molecular weight (Mr). Corresponding accession numbers were obtained by performing BLAST search in the TIGR database (http://rice.plantbiology.msu.edu/analyses_search_blast.shtml). High bulk represents the bulk of RILs possessing high root penetration ability, and low bulk represents the bulk of RILs possessing low root penetration ability. Where MS is Mascot score, MP/PS is peptide matched/peptide searched, SC% is sequence coverage percentage; abundance ratios in bold fonts indicate significant at p < 0.05.

Analysis of peptide mass fingerprint data of 29 proteins against a rice proteome database (www.matrixscience.com) resulted in the identification of 25 proteins with known functions, and others were found to be unknown proteins (Table 1). Six proteins showing upregulation in the roots of Nootripathu and high root bulk over their respective comparators were identified as peroxiredoxin, protein kinase, leucine rich repeat containing protein (LRR), Ras-related protein, pathogenesis-related Bet V family protein, and 1,2-dihydroxy-3-keto-5-methylthiopentene dioxygenase (*ARD*). Proteins that showed downregulation in the roots of Nootripathu and high root bulk included a maturase k, RPM1, proteophosphoglycan, retrotransposon protein, oxidoreductase, aldo/ketoreductase family protein, chloroplast 30S ribosomal protein, MAR binding filament-like protein, PPR repeat domain containing protein and an auxin response factor.

### Co-localization analysis of DE proteins against root QTLs identified putative candidate genes controlling root growth behavior

The physical position of differentially expressed proteins was predicted based on the rice genome database (http://rice.plantbiology.msu.edu/) and was aligned against root QTLs in the rice genetic map (www.gramene.org). Of the 33 proteins that showed differences in abundance between parents and between the bulks of RILs, 28 proteins were found to be co-localized within QTLs for root growth-related traits. Four proteins, namely, leucine-rich repeat containing protein (U7), Ras-related protein (U16), pathogenesis-related Bet V family protein (U10), and 1,2-dihydroxy-3-keto-5-methylthiopentene dioxygenase (U3), were found to exhibit significant upregulation in the roots of Nootripathu and high root bulks and co-localized with QTLs related to root number, root thickness and root penetration ability (Fig. 2c; Supplementary Table 2).

### Functional validation of *OsARD4* through genetic transformation

#### Validating differential expression of *OsARD4* in the roots of IR20 and Nootripathu

A putative candidate gene encoding 1,2-dihydroxy-3-keto-5-methylthiopentene dioxygenase (*OsARD4, acireductone dioxygenase* located on chromosome 10 between 14759781 and 14757124 bp) was selected for functional validation. qRT-PCR analysis revealed that *OsARD4* transcripts were 2.7-fold higher in the roots of Nootripathu compared to those of IR20 (Fig. 2d).

#### Sequence characterization of *OsARD4*

**Figure 3:**
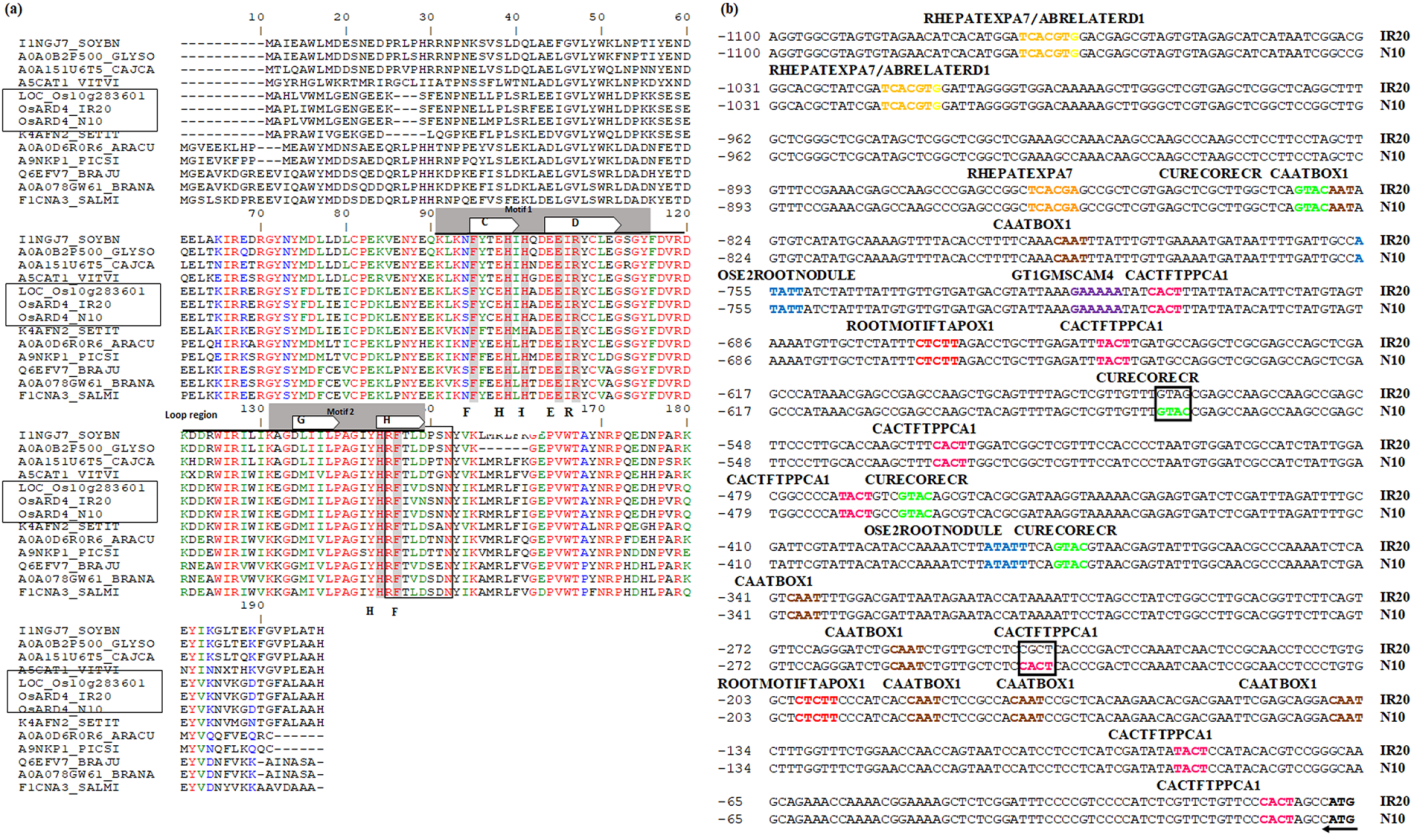
**(a)** Multiple sequence alignment of *OsARD4* homologs from rice (IR20, Nootripathu and Nipponbare), *Setaria italica* (K4AFN2); *Glycine max* (I1NGJ7); *Cajanus cajan* (A0A151U6T5); *Glycine soja* (A0A0B2P500); *Vitis vinifera* (A5CAT1); *Araucaria cunninghamii* (A0A0D6R0R6); *Picea sitchensis* (A9NKP1); *Salvia miltiorrhiza* (F1CNA3); *Brassica juncea* (Q6EFV7); and *Brassica napus* (AOAO78GW61). Bold capital letters underneath the sequences indicate the conserved metal binding amino acids in the cupins. Conserved motifs 1 and 2 are shown in grey boxes above the sequences, the open arrows show the β-strands in motifs 1 and 2, and the self-destruction canonical domain is shown in the rectangular box. **(b)** Major *cis* elements predicted in the promoters of *OsARD4* from IR20 and Nootripathu using the PLACE signal scan program. *Cis* elements are highlighted in colored fonts, and the absence of binding sites for CACTFTPPCA1 and CURECORECR in the promoter of IR20 is shown in rectangular boxes. The numbers on the left side of the sequences indicate the position of nucleotides from the initiation codon; the right side labels IR20 and N10 represent rice genotypes IR20 and Nootripathu, respectively.

PCR amplification, cloning, and sequencing of *OsARD4* (2335 bp) revealed that it encodes a 555 bp cDNA containing five exons intervened by four introns, as reported in the reference genome (www.tigr.org). It encodes a protein containing 184 amino acids similar to the *OsARD4* homolog in the database (LOC_Os10g28360). *OsARD4* homologs of IR20, Nootripathu, and Nipponbare (reference genome) shared 98.38% at the cDNA level and 95.65% at the amino acid level. Multiple sequence alignment of the *OsARD4* homologs of all three genotypes identified 12 different sequence polymorphisms (SNPs) (Supplementary Fig. 3a). Of the 12 SNPs, 6 were specific to landrace Nootripathu, and 3 were specific to IR20. Of the 6 SNPs specific to Nootripathu, 5 resulted in changes at the amino acid level (15^th^, 24^th^, 95^th^, 103^rd^ and 143^rd^ positions) (Supplementary Fig. 3b). Conserved domain analysis showed the presence of conserved motifs, *viz*., the cupin domain, metal binding residue, and destruction box motif RxxLxxxN (Fig. 3a). The 95^th^ amino acid (Y to C) variation in Nootripathu was found in the β-strand, while the 103^rd^ amino acid (F to L) variation was found in the loop region. Analysis of the secondary structures of *OsARD4* in IR20, Nootripathu, and Nipponbare revealed the presence of disorder of 13% in Nootripathu, 16% in IR20, and 15% in Nipponbare (Supplementary Fig. 4). A single amino acid variation at the 15^th^ position (K to R) was found to contribute to the disorder. The phylogenetic tree constructed using ARD4 protein sequences of 37 plant species, including IR20, Nootripathu and Nipponbare, was divided into five major clusters (Supplementary Fig. 5). All the seven rice homologs formed a separate clade and shared significant similarity with other grass family members (Supplementary Fig. 5). The *ARD4* homologs of pulses formed a separate clade, while *M. acuminata* formed an outer clade.

#### *In silico* analysis of promoter elements of *OsARD4* in IR20 and Nootripathu

Cloning and sequencing of the 1 kb upstream region of *OsARD4* revealed differences between the promoters of IR20 and Nootripathu (Fig. 3b). Two root-specific elements (RSEs), *viz*., ROOTMOTIFTAPOX1 and OSE1/2ROOTNODULE, root hair-specific element (RHE) RHERPATEXPA7, mesophyll expression module 1 (mem1) CACTFTPPCA1, and two other elements, CAATBOX1 and GTGANT10, were found to be abundant in the promoters of both the genotypes. Apart from the *cis* elements determining root-specific expression, other basal regulatory elements, such as TATA-Box, CAAT-Box, biotic or abiotic stress-responsive elements (ABRE, CGTCA, LTRECOREATECOR15, MYBCORE, and WRKY), green tissue-specific regulatory elements (G-Box, GT1-motif and I-box), and copper and oxygen-responsive element CURECORECR, were also present in both genotypes. The promoter of *OsARD4*_Nootripathu_ was found to contain eight binding sites for CURECORECR, whereas IR20 had only six elements. Similarly, the *OsARD4*_Nootripathu_ promoter was found to contain 17 binding sites for CACTFTPPCA1, whereas 16 sites were observed in *OsARD4*_IR20_ (Fig. 3b).

#### Generation and characterization of transgenic ASD16 rice lines engineered with *OsARD4*_Nootripathu_

**Figure 4:**
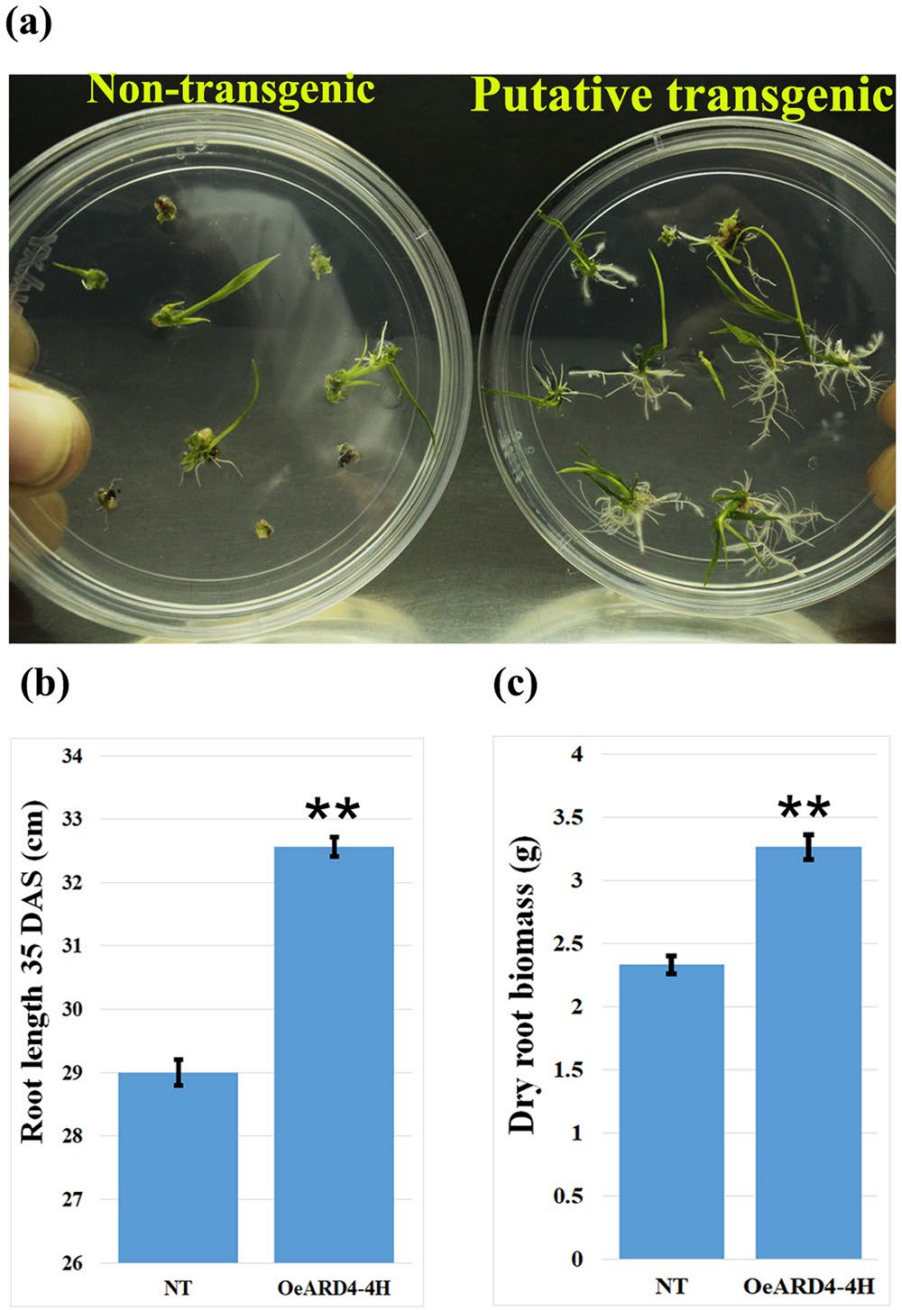
**(a**) Putative transgenic ASD16 plants exhibiting early and rapid root development compared to non-transgenic ASD16 within 5 days after transferring to rooting medium maintained at 25 °C with 16 h light/8 h dark cycles. (**b**,**c)** Represent root length and root biomass (35 days old) in non-transgenic ASD16 and a transgenic event OeARD4-4H exhibiting the highest overexpression of the transgene. Each data point is the mean of three replicates. **Indicates statistical significance at P < 0.01.

Full-length cDNA encoding *OsARD4* was isolated from Nootripathu, cloned in pCAMBIA1301 and engineered in shallow-rooted *indica* rice variety ASD16 through Agrobacterium-mediated transformation, which resulted in the generation of five putative transgenic events, *viz*., OeARD4-1A, OeARD4-2B, OeARD4-3C, OeARD4-4H, and OeARD4-5E (Supplementary Figs 6 and 7). Under each event, multiple plants were regenerated and named using alphabets suffixing the event number. Transgenic plants exhibited early initiation of rooting, i.e., within 5 days after transferring to rooting media^16^, compared to non-transgenic ASD16 (Fig. 4a). Successful integration of the transgene cassette was confirmed through PCR analysis using primers specific to the CaMV35S promoter, marker (*hpt*), and GUS reporter gene (Supplementary Fig. 6i,j), and expression of the transgene cassette was confirmed through histochemical staining (Supplementary Fig. 6f). Southern hybridization analysis revealed that all the lines have integration of multiple copies of the transgene (Supplementary Fig. 6k). Transgenic lines were found to possess higher root volume compared to non-transgenic ASD16 (Supplementary Fig. 6h).

T_1_ progenies of all five transgenic events were germinated in petri plates and found to be superior to those of the non-transgenic counterpart in their root growth characteristics (Supplementary Fig. 7a–c). All the transgenic lines were found to possess higher root numbers and root lengths compared to their non-transgenic counterpart, with varying levels of significance (Supplementary Fig. 7b,c). All transgenic lines were found to possess a significantly higher abundance of *OsARD4* transcripts, except for OeARD4-3C, for which the level of overexpression was not significantly higher than non-transgenic ASD16 (Supplementary Fig. 7d). Among the five transgenic events, OeARD4-4H, which exhibited maximum overexpression of the transgene, an increased number of roots and an increased primary root length, was chosen for further characterization. Thirty-five day old seedlings of OeARD4-4H were found to have 12% higher root length and 39% higher root biomass compared to their non-transgenic counterpart (Fig. 4b,c).

#### Overexpression of *OsARD4* promoting the root growth pattern

**Figure 5:**
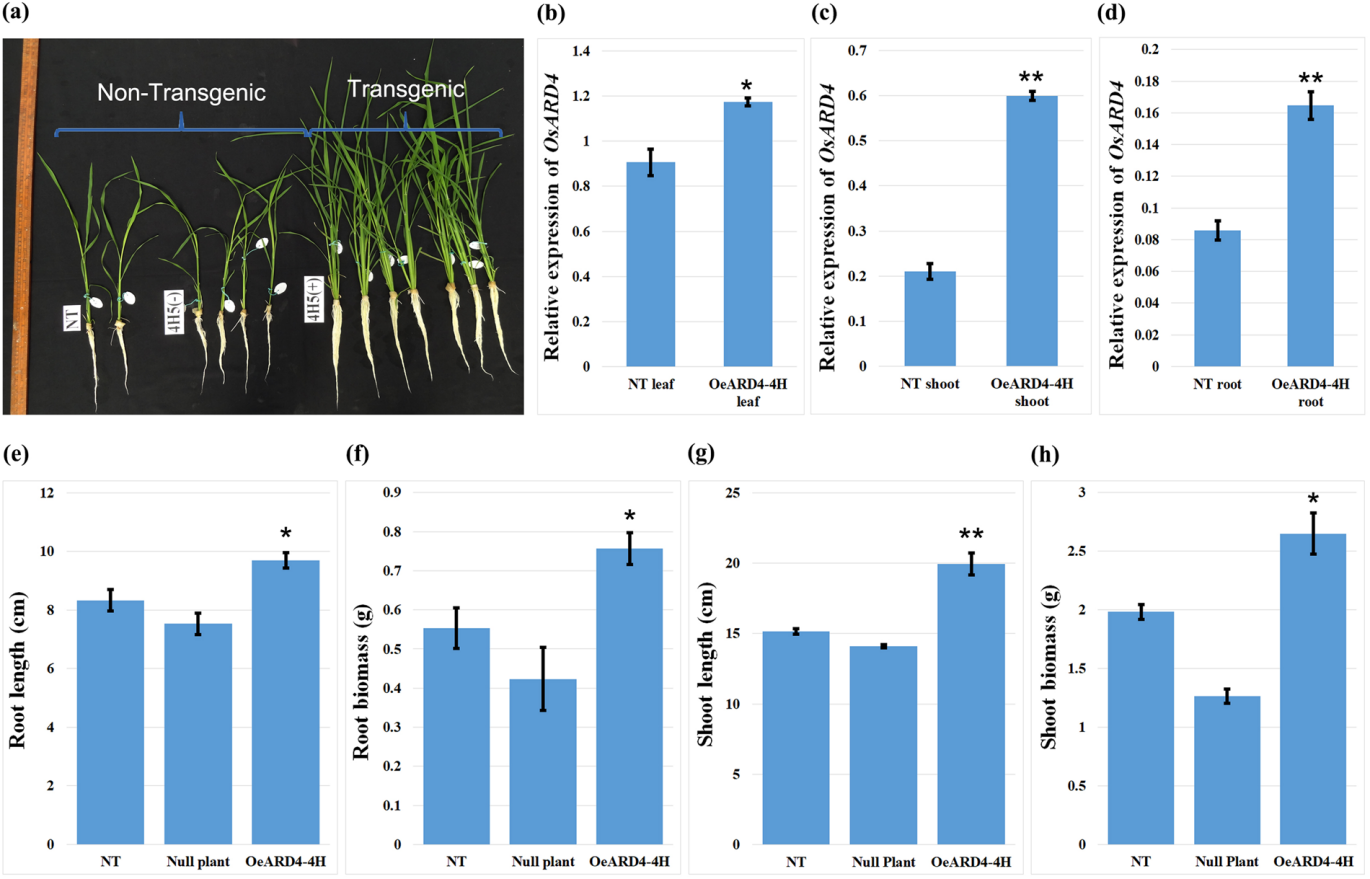
Effect of *OsARD4* on root and shoot growth characteristics of ASD16. **(a)** Transgene-positive plants of OeARD4-4H showing vigorous shoot and root growth compared to non-transgenic ASD16 under hydroponic conditions. Relative expression of *OsARD4* transcripts in leaf **(b)**, shoots **(c)** and roots **(d)** of non-transgenic and transgenic (OeARD4-4H) ASD16 plants. **(e**–**h**) Represent root length (**e**), root biomass (**f**), shoot length (**g**) and shoot biomass (**h**) in non-transgenic and transgenic ASD16 (OeARD4-4H) plants. Each data point is the mean of three replicates. *Indicates significance at P < 0.05, and **Indicates significance at P < 0.01.

Evaluation of T_2_ progenies of OeARD4-4H revealed that overexpression of *OsARD4* was found to promote overall growth of seedlings (Fig. 5a). *OsARD4* was expressed constitutively in leaf, shoot and root tissues of non-transgenic ASD16, and its overexpression was confirmed in OeARD4-4H (Fig. 5b–d). OeARD4-4H was found to have an ≈17% increased root length (Fig. 5e), an ≈36% increased root biomass (Fig. 5f), an ≈32% increased shoot length (Fig. 5g), and an ≈34% increased shoot biomass (Fig. 5h) over its comparators, namely, non-transgenic ASD16 and null plants during the seedling stage, but the differences in shoot characteristics and yield attributes between OeARD4-4H and its non-transgenic comparators were not significant at maturity (Supplementary Fig. 8a–d).

**Figure 6:**
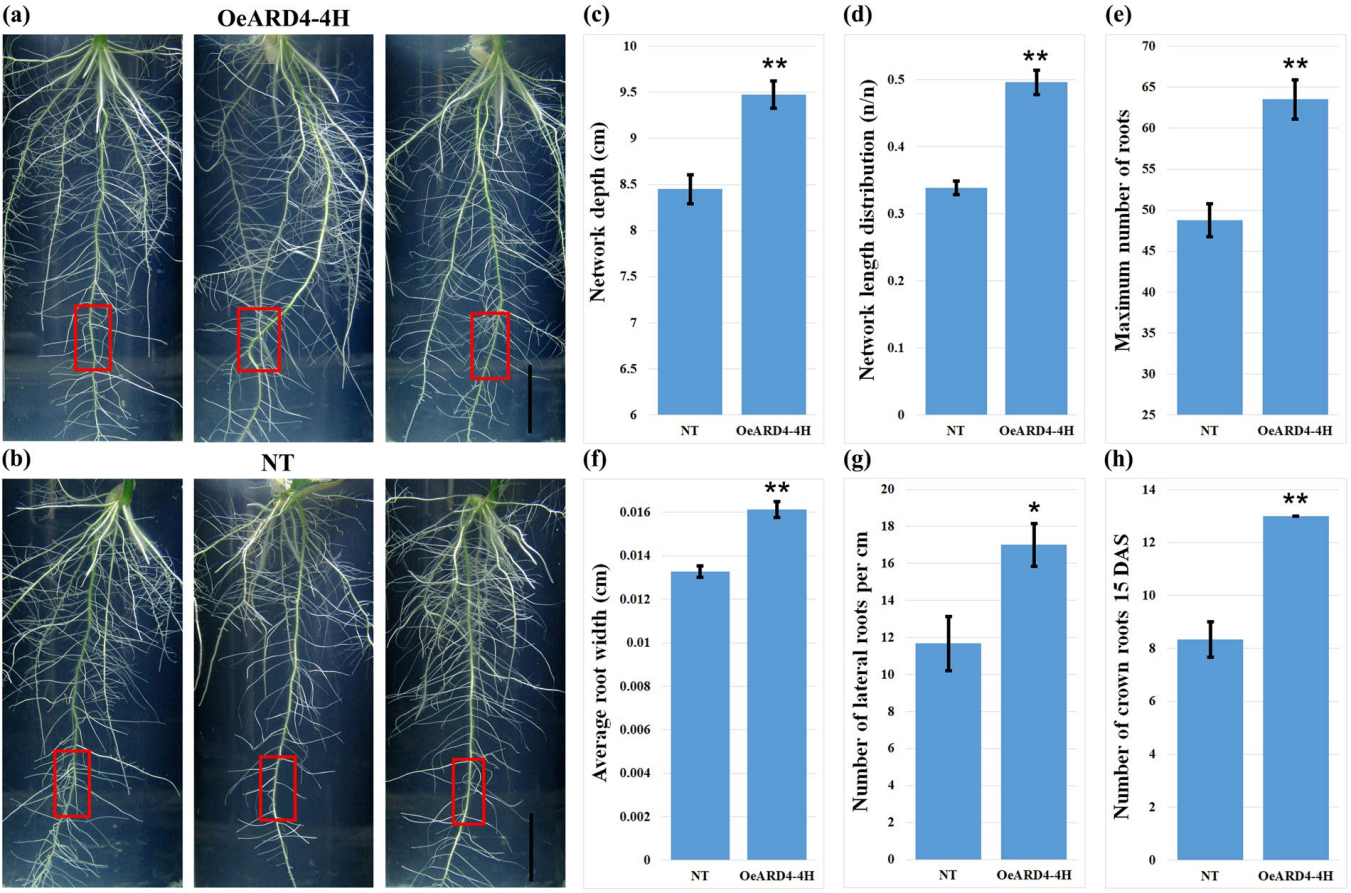
Phenotyping of root growth traits in transgenic **(a)** and non-transgenic **(b)** ASD16 plants under *in vitro* conditions; scale bar = 2 cm. (**c**–**h)** Represent the effect of overexpression of *OsARD4* on various root system architecture-related traits estimated using GIA Roots software (each value is an average of 4 plants). **Indicates statistical significance at P < 0.01, and *Indicates statistical significance at P < 0.05; red boxes indicate the 2 cm length used for recording the number of lateral roots.

**Figure 7:**
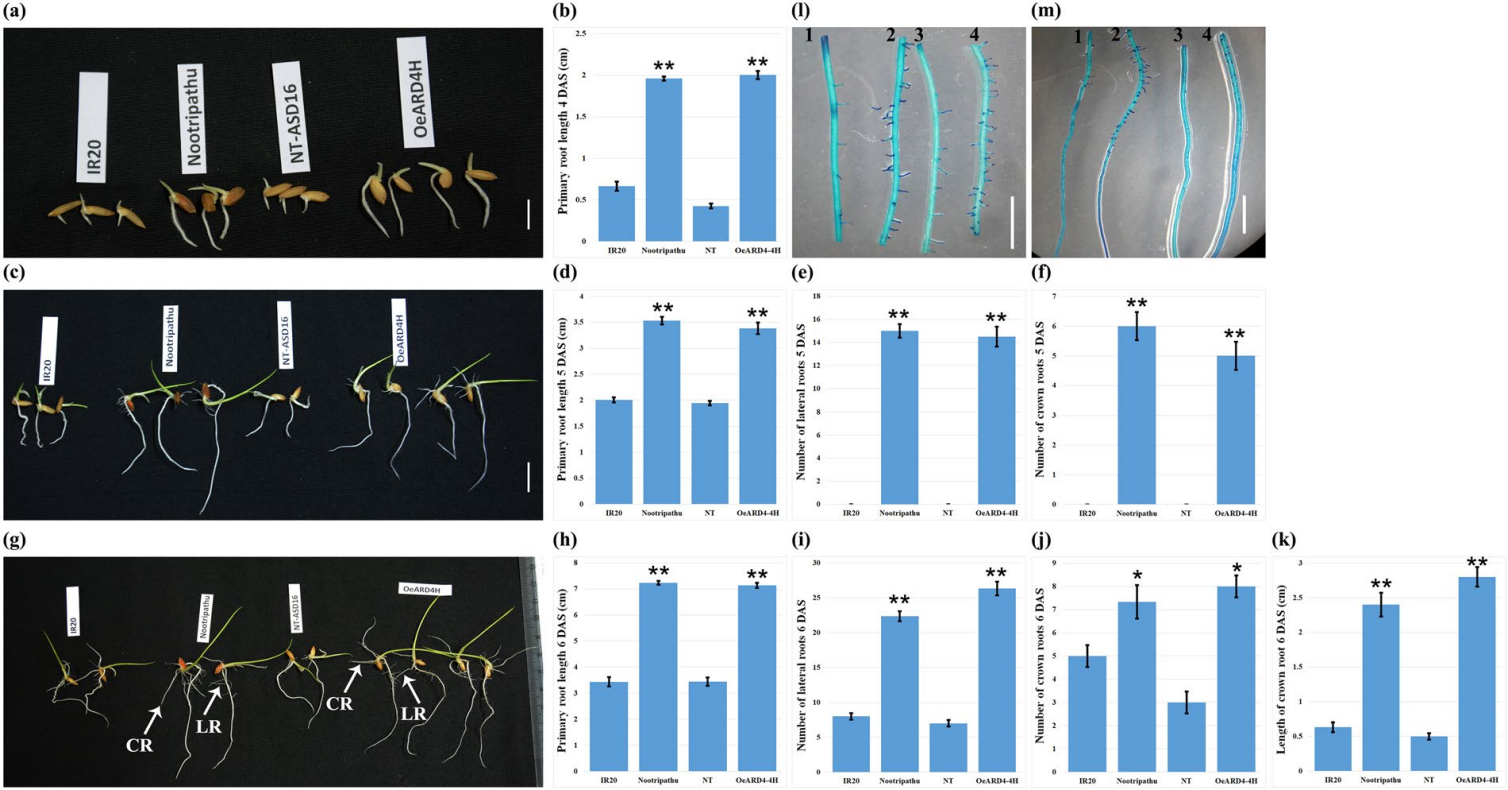
**(a)** Germinated seedlings of IR20, Nootripathu and non-transgenic and transgenic ASD16 (OeARD4-4H) at 4 days after sowing (4DAS); scale bar = 1 cm. **(b)** Length of primary roots in seedlings of IR20, Nootripathu and non-transgenic and transgenic ASD16 at 4 DAS. **(c)** Root morphology of 5-day old seedlings for IR20, Nootripathu and non-transgenic and transgenic ASD16 plants. scale bar = 1 cm. (**d**–**f)** represent the length of primary roots (**d**), number of lateral roots (**e**) and number of crown roots (**f**) in seedlings of IR20, Nootripathu and non-transgenic and transgenic ASD16 at 5 days after sowing. **(g)** Root morphology in 6-day old seedlings of IR20, Nootripathu and non-transgenic and transgenic ASD16 plants; CR, crown roots, and LR, lateral roots. (**h**–**k)** Represent the length of primary roots (**h**), the number of lateral roots (**i**), the number of crown roots (**j**) and the length of crown roots (**k**) on 6 DAS. **(l)** Distribution pattern of lateral roots and lateral root primordia in the top 2 cm and **(m)** in the bottom 3 cm of roots; scale bar = 0.5 cm. Each data point is the mean of three replicates; *Indicates significance at P < 0.05, and **Indicates significance at P < 0.01 (Significance was tested using One-way ANOVA between IR20 vs Nootripathu and non-transgenic ASD16 (NT) vs OeARD4-4H).

**Figure 8:**
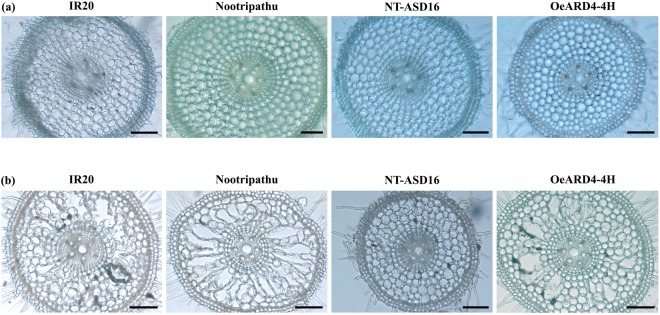
Microscopic view of cross sectioned primary roots in IR20, Nootripathu, NT-ASD16 and OeARD4-4H seedlings on 4 DAS (**a**) and 6DAS **(b)**. Scale bar = 100 µM.

Digital imaging of the root system architecture in T_3_ progenies of OeARD4-4H and *in silico* analysis of root images revealed that transgenic and non-transgenic ASD16 plants differed significantly in the total number of roots and in network length (Fig. 6a–h). Transgenic ASD16 lines were found to possess higher numbers of crown roots and lateral roots than non-transgenic ASD16. *In vitro* germination studies in IR20, Nootripathu and non-transgenic and transgenic (T_4_) ASD16 revealed that seeds of transgenic (OeARD4-4H) ASD16 and Nootripathu exhibited early germination (3^rd^ day after sowing) and faster elongation of the radicle over their respective comparators, i.e., non-transgenic ASD16 and IR20 (Fig. 7a). OeARD4-4H and Nootripathu were found to possess significantly longer primary roots than non-transgenic ASD16 and IR20 (Fig. 7b). On 5 DAS, Nootripathu and transgenic ASD16 (OeARD4-4H) were found to exhibit significant differences in primary root length and initiation of primordia, leading to the development of crown roots and lateral roots over their comparators (Fig. 7c–f). On the 6^th^ day after sowing, Nootripathu and transgenic ASD16 exhibited a significantly improved root system architecture measured in terms of the primary root length (Fig. 7g,h), the number of lateral roots (Fig. 7i–m), the number of crown roots (Fig. 7j) and the length of crown roots (Fig. 7k). Further, roots of Nootripathu and transgenic ASD16 were found to exhibit rapid development of aerenchyma compared to IR20 and non-transgenic ASD16 (Fig. 8).

## Discussion

The root system plays an important role in the absorption and translocation of water and nutrients from soil in addition to providing anchorage to plants. During drought conditions, roots enable the plants to avoid drought by maintaining a high internal water status through increased water uptake from the deeper layers of the soil^17^. Rice, one of the staple cereal food crops, is extremely sensitive to drought, but some of the upland rice genotypes are reported to be drought tolerant^18^. Root traits, *viz*., the number of lateral roots, deep rooting, and accelerated elongation towards moisture gradients in upland rice cultivars, make them more adaptable to water-limited environments^6,19^. It is hypothesized that the introduction of deep rooting characteristics of upland rice genotypes into shallow-rooted lowland rice cultivars will help in improving the drought tolerance ability.

Progress in developing high yielding rice genotypes with deep-rooting and enhanced drought avoidance/tolerance is limited due to the genetic complexity of root trait(s) and the inherent difficulty in phenotyping. Recent advancements in molecular genetics and genomics enabled the mapping of robust QTLs controlling various root growth-related traits using bi-parental mapping populations, *viz*., IR64/Azucena, Kalinga III/Azucena, IR64/Kinandang Patong and available natural genetic variations^2,6,8^. Over several years of intensive research on the molecular basis of root growth behavior in rice, only a few genes, namely, *PSTOL1*, which regulates enhanced P uptake through extensive roots under low P conditions^20^, *DRO1*, which regulates root angle^2^, and *OsORC3*, which regulates lateral root development^21^, have been characterized at a functional level. Despite advancements in genomics and related bioinformatics, functional genomics of root growth behavior in rice remains unexplored. In this study, gene expression profiling, bioinformatics, genetic transformation, and precision phenotyping procedures were combined to unravel the molecular basis of deep root growth behavior in an upland rice cultivar, Nootripathu.

Landrace Nootripathu was found to possess higher numbers of roots and increased root length and thickness compared to IR20. The number and size of the cells in the root meristem and root cap characteristics have been attributed to a more rapid emergence of the radicle, to faster elongation of the roots and to survival of the roots under stress conditions^22,23^. Larger root caps are said to limit the rapid elongation of roots because of the accumulation of larger amounts of basipetally transported growth inhibitors in the larger root caps than in the smaller root caps, thus preventing rapid elongation of the roots^23,24^. The number of dividing cells in root tips is associated with an accelerated root elongation rate in maize^24^. In this study, landrace Nootripathu was found to possess relatively shorter root caps, a higher number of dividing cells in the root meristem, and larger cells compared to IR20. These adaptive features would have enabled Nootripathu to exhibit faster germination, rapid elongation of the radicle, and early development of the crown/lateral roots.

Survival of plants during drought largely depends on the ability of roots to penetrate the hardpans in soil, thereby reaching water in deeper layers to maintain a high internal water status. Genetic variation for RPA and root thickness has been reported earlier in rice^25,26^. In this study, landrace Nootripathu was found to possess higher number of roots, increased root length, and increased thickness to exhibit higher RPA (22%) through the wax petrolatum layer compared to IR20 (4.6%). RILs exhibited continuous genetic variation for the number of roots, the primary root length and number, and RPA (Fig. 1f,g; Supplementary Fig. 2), which enabled grouping of extreme RILs as low root bulk and high root bulk.

Bulked segregant analysis (BSA) is an efficient strategy for tagging traits governed by major gene(s). In this study, 2D-PAGE profiling of proteins in root tips of IR20, Nootripathu, and extreme bulks of their RILs resulted in the identification of differentially expressed proteins co-segregating with root growth behavior. It was demonstrated in *Arabidopsis* that proteomics can be a powerful strategy to identify genes/proteins involved in primary root development and elongation^27^. Proteins involved in carbohydrate metabolism, hormonal synthesis and responses, signaling, ROS scavenging, energy usage, and membrane integrity were found to be upregulated in root tips of landrace Nootripathu and high root bulk. Upregulation of genes involved in turgor maintenance, cellular integrity, reactive oxygen scavenging, cell signaling, carbohydrate metabolism, and cell wall biosynthesis has already been reported in roots of drought tolerant genotypes in rice^28^, soy bean^29^ and Vigna^30^. Proteins such as LRR (leucine rich repeat proteins), Ras proteins, annexins, hexokinase and MAPK were abundantly expressed in roots of Nootripathu and high root bulk than in compared to their low root counterparts. LRR/extensin was reported to be involved in establishing root hair polarization and tip growth by physically connecting the cell wall and the plasma membrane^31^. Ras proteins are small GTPases that function as signaling nodes in regulating cell growth, proliferation and differentiation and are involved in auxin-mediated root growth^32^.

It has been reported that annexins are associated with the polarization of root apical cells and elongation^33,34^, and a specific annexin (*AnxAt*2) was shown to be involved in root hair elongation and lateral root development in Arabidopsis^35^. The abundant expression of hexokinases (HXKs) in the root tips of Nootripathu may be associated with increased sugar signaling and, thereby, may promote meristematic and cell division activities resulting in enhanced root growth, as demonstrated in Arabidopsis^36^. Similarly, mitogen-activated protein (MAP) signaling cascades mediated by kinases are known to be crucial for root hair elongation^37,38^. Advancements in gene expression profiling and bioinformatics accelerated candidate gene discovery through genomics approaches. Putative candidate genes underlying complex quantitative traits can be dissected out through the combined analysis of gene expression data and genetic map data to map differentially expressed genes against related QTLs. In this study, the alignment of differentially expressed proteins against QTLs for root traits led to the identification of putative candidate genes/proteins showing up/downregulation in roots of Nootripathu as well as high root bulk over their respective comparators and co-localized within QTLs for root growth-related traits.

One such candidate protein encoding 1,2-dihydroxy-3-keto-5-methylthiopentene dioxygenase or *acireductone dioxygenase* (*OsARD4*) that showed upregulation in roots of Nootripathu and high root bulk was found to be co-localized with QTLs for root length, root thickness, and root penetration ability. *Acireductone dioxygenases* are known to regulate the methionine salvage pathway, ethylene biosynthesis and polyamine biosynthesis^39^. Specific overexpression of *OsARD4* in roots of landrace Nootripathu and high root bulk indicated its strong functional relevance in the promotion of the root growth pattern.

The *OsARD4* homologs in IR20 and Nootripathu shared significant homology with reference Nipponbare at both the nucleotide and amino acid levels. The presence of conserved motifs and the metal binding residues along with the destruction box motif RxxLxx(x)N in the *OsARD4* homolog revealed its functional similarity to other ARD proteins. *OsARD4* homologs of IR20, Nootripathu and Nipponbare differed significantly in their structural disorders. The disordered regions indicate that the weakest region of the helix is in a protein secondary structure. Amino acids in these regions are predicted to be unstructured. The analyses showed that there is an increased disorder percentage in IR20, which may lead to functional differences in the protein. However, further experiments are required to prove this. Variation in two amino acids at the 95^th^ and 103^rd^ positions and differences in the structural disorder among the *OsARD4* homologs of IR20 and Nipponbare may have some implications for its functional efficiency.

Cloning, sequencing and analysis of promoter regions of *OsARD4* in IR20 and Nootripathu revealed the presence of root-specific modules, such as the ROOTMOTIFTAPOX1, OSE1/2 ROOTNODULE and RHERPATEXPA7 (RHEs - root hair-specific cis elements) elements, CACTFTPPCA1 (mesophyll expression module 1 - mem1), CAATBOX 1, and GTGANT10, as in other root-specific genes in rice^40^. A combination of RSEs and mem1 elements in root-specific promoter sequences is predicted to have synergistic interactions in regulating root-specific expression^41^. The over-representation of CACTFTPPCA1 (mem1) and CURECORECR (oxygen and copper-responsive element) elements in the promoter of *OsARD4*_Nootripathu_ could be attributed to differences in the expression of *OsARD4* in contrasting rice genotypes, which needs validation using marker gene constructs driven by the *OsARD4* promoters of IR20 and Nootripathu.

Agrobacterium-mediated transformation of ASD16 using pCAMBIA1301 harboring *OsARD4*_Nootripathu_ resulted in the generation of 5 putative transgenic events. All transgenic lines showed early initiation of rooting and proliferous branching of roots, indicating a possible influence of *OsARD4* on root development (Fig. 4a). Analysis of T_1_ progenies of all transgenic lines led to the identification of OeARD4-4H possessing the maximum overexpression of transgene *OsARD4*, the highest primary root length and the maximum number of roots for further characterization. Overexpression of *OsARD4* was found to exhibit profound promotional effects on the overall growth and development of seedlings (Fig. 5a). Transgenic plants were found to possess significantly longer roots and a higher root and shoot biomass compared to non-transgenic ASD16 during the early stages of crop growth (Fig. 5e–h). However, transgenic and non-transgenic plants became on par in their shoot characteristics (Supplementary Fig. 8a,b) and yield-related traits (Supplementary Fig. 8c,d) during the later stages of crop growth, but the differences in root traits were maintained until maturity. Seedling vigor is an important trait determining early establishment and weed competitiveness of the main crop under upland rice cultivation^42^, and Nootripathu is one of the traditional rice genotypes highly adapted to upland aerobic cultivation. Overexpression of *OsARD4* in ASD16 might have allowed the lowland rice variety ASD16 to acquire such drought-adaptive traits exhibited by Nootripathu.

To understand the role of *OsARD4* in root development, T_3_ progenies of OeARD4-4H were subjected to thorough phenotypic evaluation of root characteristics. Digital imaging of the root system architecture and image analysis clearly demonstrated that transgenic plants were found to possess an increased number of lateral roots and increased network length compared to the non-transgenic plants. Root systems with more lateral roots are reported to be agronomically significant for providing maximum physical support and better absorption^2^. Optimal distribution of lateral roots in the primary root is considered a key factor determining early vigor of the plant^43^. Early germination with longer primary roots and increased crown/lateral roots was evident in OeARD4-4H exhibiting overexpression of *OsARD4*. Development of aerenchyma in the cortex is one of the adaptive strategies to withstand water deficit conditions by reducing the metabolic cost of root cells and hydraulic conductivity^44^. Aerenchyma cells will also facilitate the rapid transport of ethylene formed during oxygen deficit conditions in rice. Microscopic examination in root tips of contrasting rice genotypes revealed that roots of Nootripathu and OeARD4-4H showed earlier and more rapid development of aerenchyma than their counterparts (Fig. 8).

As reported earlier, the ARD gene family is involved in the biosynthesis of ethylene through the methionine salvage pathway^39^. Hormones such as ABA, ethylene, and auxin serve as key signals in shoot/root communication under normal and stress conditions^45^. Specifically, ethylene is reported to be the key element in regulating aerenchyma formation, adventitious root growth, and internodal elongation in rice^46,47^. ARD is an effector protein for the functioning of G signaling proteins and was found to be essential for cell division in Arabidopsis^48^. Overexpression of *OsARD4* might have contributed to the alteration of several GA-mediated processes, *viz*., cell division, carbohydrate metabolism, and cell elongation, leading to an enhanced seedling growth rate, early development of lateral roots, and increased root biomass in the transgenic line. This study has clearly demonstrated the association between the expression levels of *OsARD4* and the regulation of root growth patterns in rice. Thus far, only a few functional genes regulating root growth characteristics in rice have been reported. This study has generated improved knowledge of the molecular basis of root growth and development in rice. The evaluation of transgenic lines under drought stress conditions will shed more light on the putative role(s) of *OsARD4* in modulating drought responses in rice through the regulation of root growth characteristics. More research on analyzing the effects of amino acid/promoter variations on the function and expression of *OsARD4* will allow the precise manipulation of root growth characteristics in rice through molecular breeding.

## Materials and Methods

### Genetic materials used

Two contrasting rice genotypes, namely, shallow-rooted drought-susceptible IR20 and deep-rooted drought-tolerant landrace Nootripathu^14^, and a set of RILs (F_8_ generation) developed between them were used in this study.

### Plant growth conditions

All hydroponic cultures and PVC pipe experiments were carried out under greenhouse conditions, i.e., at 28 ± 2 °C with ≈12 h light/12 h dark photoperiod conditions with a relative humidity of 80 ± 5%, at Tamil Nadu Agricultural University, Coimbatore, India. Hydroponically grown plants were maintained in trays filled with Yoshida solution^49^ (pH: 4.5–5.0). PVC pipes were filled with field clay soil (collected from Paddy Breeding Station, Tamil Nadu Agricultural University located at GPS coordinates of 11°00′N and 76°54′E; 426 m above sea level) and a coir pith mixture (1:1) along with the required amount of fertilizer [0.5 g of (NH_4_)_2_SO_4_, 0.04 g of muriate of potash (KCl), and 0.04 g of single superphosphate (SSP)] per kg of soil:coir pith mixture.

### Characterization of root traits in IR20 and Nootripathu

Seedlings of IR20 and Nootripathu were raised in cylindrical PVC pipes (1 m long) and allowed to grow for up to 45 days. Each genotype was replicated thrice by maintaining one plant per tube. Plants were pulled out on the 46^th^ day, and observations on the total number of roots per plant, root length (mm) and root thickness (µm) were recorded. One-Way ANOVA was performed to test the mean difference. To study the anatomical variations in roots of IR20 and Nootripathu, seeds were germinated in petri plates, and root tips (2 cm from the tip) were excised on the 5^th^ day after germination and fixed in FAA (50% ethyl alcohol:acetic acid:formalin = 90:5:5) as described earlier^50^. Dehydrated root segments were embedded in wax, and transverse sections (5 µm thickness) were taken in both divisional and elongation zones using a semi-automatic rotary microtome (Model No. 1010-SMT-006, Microtomes India, New Delhi, India) and stained with safranin-O/fast green (0.1% safranin, 0.01% fast green and 1% acetic acid). Stained specimens were viewed on a phase contrast microscope (Motic B1–223ASC model, Wedgwood AV Ltd, United Kingdom). The number of cells, length/width of cells, root cap length, and root thickness were measured using an ocular micrometer. Duncan’s multiple range test (DMRT) at a 0.05 probability level was used to group means. Similarly, the length and width of matured cortical cells were measured using hand sections made in the primary roots (4–5 cm from the tip) of 35 day old seedlings grown under greenhouse conditions.

### Evaluation of RILs of IR20 and Nootripathu for root morphology and penetration ability

Seeds of IR20, Nootripathu, and a subset of their RILs were germinated in petri plates and used for recording observations on root growth patterns. The length of primary roots was measured in five individual seedlings of all genotypes, and means were tested using one-way ANOVA. The root penetration ability of IR20, Nootripathu, and their RILs was assessed using a wax petrolatum layer (WPL) system simulating hardpans in soil formed during drought, as described earlier^26^. WPLs measuring 12 cm in diameter and 3–4 mm in thickness were prepared by mixing molten paraffin wax and petroleum white jelly at the ratio of 2:1 (w/w). Each WPL was placed inside a plastic pot with their bottoms removed, and the layer was supported by a perforated plastic crater. Pots were filled with soil and coir pith (1:1) to raise seedlings of contrasting rice genotypes and their RILs and were maintained under greenhouse conditions. Each genotype was replicated thrice, and observations on various root traits, *viz*., the total number of roots and number of roots penetrating the WPL, were made on 45 DAS. RPA was calculated as the percentage of roots that had penetrated the wax layer relative to the total number of roots. One-Way ANOVA was performed, and means were grouped using Fisher’s least significant difference (LSD). RILs exhibiting a contrasting root growth pattern were selected for further studies.

### 2D-PAGE analysis of root proteins in IR20, Nootripathu and bulks of extreme RILs

#### Sampling of root tissues

Seedlings of rice genotypes, *viz*., IR20, Nootripathu, and RILs exhibiting contrasting root growth behavior (3 RILs possessing low roots and 3 RILs possessing high roots), were established under hydroponic conditions. Root tissues (2 cm from tip) were collected from 3 individual plants of each genotype on the 15^th^ day after germination and constituted one replication. Likewise, three independent biological replications were collected and used for further studies.

#### Protein extraction and bulking

Proteins were isolated from the root tips of individual genotypes as described earlier^51^, quantified through the Bradford method and stored at −80 °C. Equal amounts of protein were taken from 3 RILs possessing profuse rooting and constituted as high root bulk, and similarly, low root bulk was constituted by bulking an equal amount of protein isolated from roots of RILs possessing relatively poor root growth behavior. Proteins isolated from the roots of parents (IR20 and Nootripathu) and extreme bulks of the RILs were used for 2D-PAGE analysis.

#### Identification of differentially expressed proteins

Equal amounts of protein (150 μg) from IR20, Nootripathu, and bulks of extreme RILs were separated by 2D-PAGE. IPG strips (17 cm length and pH 4–7) were rehydrated with approximately 150 µg of protein and subjected to isoelectric focusing (IEF) using Multiphor II (M/s. GE Health Care Ltd., USA) at 500 V for 1 h, followed by 1000 V for 1 h and 3000 V for 12–14 h. After IEF, proteins were separated using SDS–PAGE (12% polyacrylamide gels) and visualized by the silver staining method. Triplicate gels were obtained from three independent replications of each sample.

#### Protein identification through MALDI-TOF

Silver stained gels were scanned using a Labscan – Image Scanner III (GE Healthcare, USA). The relative abundance of protein spots was quantified and used for calculating the abundance ratio of proteins for IR20 *vs*. Nootripathu and low root bulk *vs*. high root bulk. Proteins exhibiting upregulation (>1.5-fold) or downregulation (<1.5-fold) between root tissues of parents (IR20 and Nootripathu) were identified, and the expression level of the corresponding proteins between contrasting bulks of RILs was calculated. The approximate molecular mass of differentially expressed proteins was determined by co-electrophoresis of standard protein markers (Amersham Biosciences, USA), and the p*I* of the proteins was determined based on the migration of the protein spots on 17 cm IPG (pH 4–7) strips. Selected spots were excised from preparative gels (stained with Coomassie brilliant blue) and subjected to MALDI-TOF analysis (Shrimpex Biotech Services Pvt. Ltd., Chennai, India). The peptide mass fingerprint data obtained were searched against rice proteome databases using MASCOT (www.matrixscience.com).

#### Co-localization analysis of differentially expressed proteins against QTLs for root traits

The physical position of differentially expressed proteins was assigned using the rice genome sequence database (http://rice.plantbiology.msu.edu/) and was aligned against QTLs for various root growth-related traits reported in the Gramene database (http://archive.gramene.org/qtl/) and in the public domain^52^. Differentially expressed proteins located within the boundary of QTLs for root traits such as root length, root density, root thickness, root penetration ability and deep rooting were shortlisted for further characterization.

### Functional validation of putative candidate gene *OsARD4* through genetic transformation

#### Validating the differential expression of *OsARD4* in roots of IR20 and Nootripathu

Root (2 cm from tip) tissues were collected from 15-day old hydroponically grown plants of IR20 and Nootripathu and used for the extraction of total RNA as per the manufacturer’s protocol (Biobasic Inc., Canada). DNAse-treated total RNA (approximately 1 µg) was converted to single-stranded cDNA using a Transcriptor High Fidelity cDNA Synthesis Kit (Roche, Germany), and an equal amount of single-stranded cDNA was used for qRT-PCR analysis (Step One Plus, Applied Biosystems, USA). No template controls (NTC) were maintained to rule out cross contamination. Each sample was biologically replicated in triplicate, and the relative abundance of mRNAs was calculated through the comparative Ct (ΔΔCt) method^53^ using ubiquitin as the endogenous control. The significance between means was tested using one-way ANOVA followed by the Tukey HSD test^54^. Information on primer sequences used for the amplification of *OsARD4* transcripts is given in Supplementary Table 3.

#### Sequence characterization of *OsARD4* in IR20 and Nootripathu

Full-length cDNA encoding *OsARD4* (LOC_Os10g28360) was amplified through RT-PCR using gene-specific primers (Supplementary Table 3), cloned in the pTZ57R/T vector using an InsTAclone PCR Cloning Kit (Thermo Scientific, USA) and sequenced (SciGenom Laboratory, Cochin, India). Nucleotide and translated amino acid sequences of *OsARD4* were used for BLASTn/BLASTp search against cDNA/protein sequences in the NCBI database (http://blast.ncbi.nlm.nih.gov/Blast.cgi). Multiple sequence alignment of nucleotide and amino acid sequences of *OsARD4* was carried out using the CLUSTALW tool in BioEdit software. The presence of conserved/catalytic domains in the deduced amino acid sequence was analyzed using the ExPASy tool (http://www.expasy.ch/tools/dna.html). Secondary structure prediction and disorder prediction were carried out using ExPASy (Protein Homology/analogy Recognition Engine V 2.0) Phyre 2 software using hidden Markov models via HHsearch (http://www.sbg.bio.ic.ac.uk/phyre2)^55^. The genetic relatedness of *OsARD4* among 37 plant species, including IR20, Nootripathu, and Nipponbare, was studied using the UPGMA method. The evolutionary distances were computed by the Poisson correction method using MEGA7^56^.

#### *In silico* analysis of promoter regions of *OsARD4* in IR20 and Nootripathu

One kilobase upstream (promoter) region of *OsARD4* was amplified from genomic DNA isolated from leaves of both IR20 and Nootripathu using promoter-specific primers, and the amplified 5′ UTR regions were cloned in the T/A vector and sequenced as described above. The presence of cis/regulatory elements was analyzed using the PLACE signal scan program.

#### Construction of plant transformation vector harboring *OsARD4*_Nootripathu_

Full-length cDNA encoding *OsARD4* was released from pTZ57R/T by *SmaI*/*XbaI* restriction digestion and eluted. Purified fragments were cloned in plant transformation vector pCAMBIA1301 under the control of the CaMV35S promoter, GUS reporter, and *nos* terminator and transformed into *E.coli. p*CAMBIA1301 harboring *OsARD4* was further mobilized into *Agrobacterium* strain LBA4404 through the freeze-thaw method^57^.

#### Developing transgenic ASD16 rice lines overexpressing OsARD4_Nootripathu_ through Agrobacterium-mediated transformation

High-yielding, shallow-rooted semi-dwarf, and tissue culture-friendly rice cultivar ASD16 was selected for overexpressing *OsARD4* through genetic transformation. Immature embryos of ASD16 were dissected out from developing panicles and co-cultivated with the *Agrobacterium* strain LBA4404 harboring pCAMBIA1301 engineered with the putative candidate gene *OsARD4*_Nootripathu_^16^. Co-cultivated immature embryos with proliferating calli were subjected to selection in MS medium containing 50 mg/l hygromycin, and putative transgenic calli were regenerated in the presence of 1 mg/ml NAA and 3 mg/ml 6-BA. Successfully regenerated plantlets were allowed to root in half MS media containing 50 mg/l hygromycin. Putative transgenic plants were transferred to a transgenic green house facility for hardening and establishment.

#### Molecular characterization of putative transgenic plants (T_0_)

Putative transgenic ASD16 plants engineered with *OsARD4*_Nootripathu_ were screened through PCR using primers specific to the promoter (CaMV35S), selectable marker (hygromycin) and reporter (GUS) genes as listed in Supplementary Table 3. Integration and expression of the transgene were confirmed through GUS histochemical staining and Southern hybridization analysis. For Southern hybridization, genomic DNA isolated from the putative transgenic plants was digested with *HindIII*, resolved on 1% agarose gel and blotted onto a positively charged nylon membrane along with genomic DNA isolated from non-transgenic ASD16 (NT) plants and hybridized using P^32^-labeled fragments of the hygromycin (*hpt*) marker gene. After undergoing hybridization, the membranes were washed, dried and exposed to X-ray film (Kodak Photo Film) overnight and developed. Histochemical staining of GUS expression was performed in roots of putative transgenic and non-transgenic ASD16 plants^58^.

#### Generation advancement and phenotypic characterization of transgenic ASD16 rice lines

Transgenic ASD16 plants were advanced up to the T_4_ generation through selfing. In the T_0_ generation, observations on the number of days taken for the initiation of rooting in the regenerating plantlets, root length, and root biomass of hardened plants before transplantation were recorded. In the T_1_ generation, progenies of all transgenic lines were germinated in petri plates, and observations on the number of roots and primary root length were recorded on the 5^th^ day after sowing. The plants were transferred to hydroponic conditions and allowed to grow for up to 35 days. Root (2 cm from tips) samples were collected from all transgene-positive plants for qRT-PCR analysis of *OsARD4*, as described earlier. Observations on root length and root biomass were also recorded in OeARD4-4H along with the non-transgenic ASD16. In the T_2_ generation, approximately 60 seeds from OeARD4-4H were germinated in petri plates along with non-transgenic ASD16. Germinated seedlings (7 days old) were transferred to a hydroponics system (trays filled with Yoshida solution) and allowed to grow for 35 days. Genomic DNA was isolated from 4–5 leaved plants and subjected to PCR analysis using *hpt* gene-specific primers. Observations on root length and root biomass were recorded in both transgenic and non-transgenic ASD16 plants. Three biological replicates of root (2 cm from tip), shoot (4 cm section above the root-shoot junction), and leaf (top 3 leaves) tissues were sampled from hydroponically grown plants of transgenic and non-transgenic ASD16 and used for total RNA extraction. The abundance of *OsARD4* transcripts was analyzed through qRT-PCR as described earlier. A set of transgene-positive plants was allowed to grow until maturity along with transgene-negative and non-transgenic ASD16 plants and used for recording observations on plant height (cm), panicle length (cm) and yield per plant (g).

#### *In vitro* phenotyping of root traits in transgenic ASD16 (T_3_)

For the *in vitro* characterization of the root system architecture (RSA), surface sterilized seeds of non-transgenic and transgenic ASD16 were inoculated into glass tubes (40 cm height and 5 cm diameter) filled with half MS media containing 0.3% Gelrite (M/s. Sigma Aldrich, USA) as a solidifying agent. The tubes were incubated at 25 ± 2 °C with a 14 h light/10 h dark photoperiod for 14 days. On the 15^th^ day, RSA was photographed using a high-resolution Nikon Digital SLR camera (by keeping the test tubes inside a transversely aligned rectangular glass tank filled with water), and RSA-related parameters were analyzed using “GIAroots” software with default settings^59^.

#### Characterization of radicle emergence, root elongation, and development of crown roots and lateral roots in transgenic ASD16 (T_4_)

T_4_ progenies of OeARD4-4H were germinated in petri dishes along with non-transgenic ASD16, IR20 and Nootripathu. Observations of the number of days taken for the emergence of radicle(s), the length of primary roots, the number of crown roots, and the number of lateral roots (at 4, 5 and 6 days after seeding) were recorded in 10 individual progenies of all four genotypes. The pattern of lateral root initiation and the length of lateral roots were recorded in roots of 8-day old seedlings after they were stained with methylene blue^21^. Anatomical variations and progression in aerenchyma development were recorded in roots of 4 and 6 day old seedlings of all four genotypes. Cross sections of developing roots (at 10–15 mm below the shoot-root junction) were taken using hand and table microtomes (M/s. Radical Scientific Equipments Ltd., Haryana, India) and visualized and photographed using an RXLr-5 Nexcope microscope (Radical Scientific Equipments Ltd., Haryana, India).

## Electronic supplementary material

Supplemntary Information
