# Supplementary material for: OsARD4 encoding an acireductone dioxygenase improves root architecture in rice by promoting development of secondary roots
Source: Sci Rep. 2018 Oct 24;8:15713. doi: 10.1038/s41598-018-34053-y (PMC6200752; doi:10.1038/s41598-018-34053-y)
Supplement: Supplementary file 1 — Supplemntary Information [file 41598_2018_34053_MOESM1_ESM.pdf]

Supplementary Information

OsARD4 encoding an *acireductone dioxygenase* improves root architecture in rice by promoting development of secondary roots

Valarmathi Ramanathan**, Hifzur Rahman**, Saravanan Subramanian, Jagadeeshselvam Nallathambi, Ashokkumar Kaliyaperumal, Sudha Manickam, Chandrababu Ranganathan and Raveendran Muthurajan*

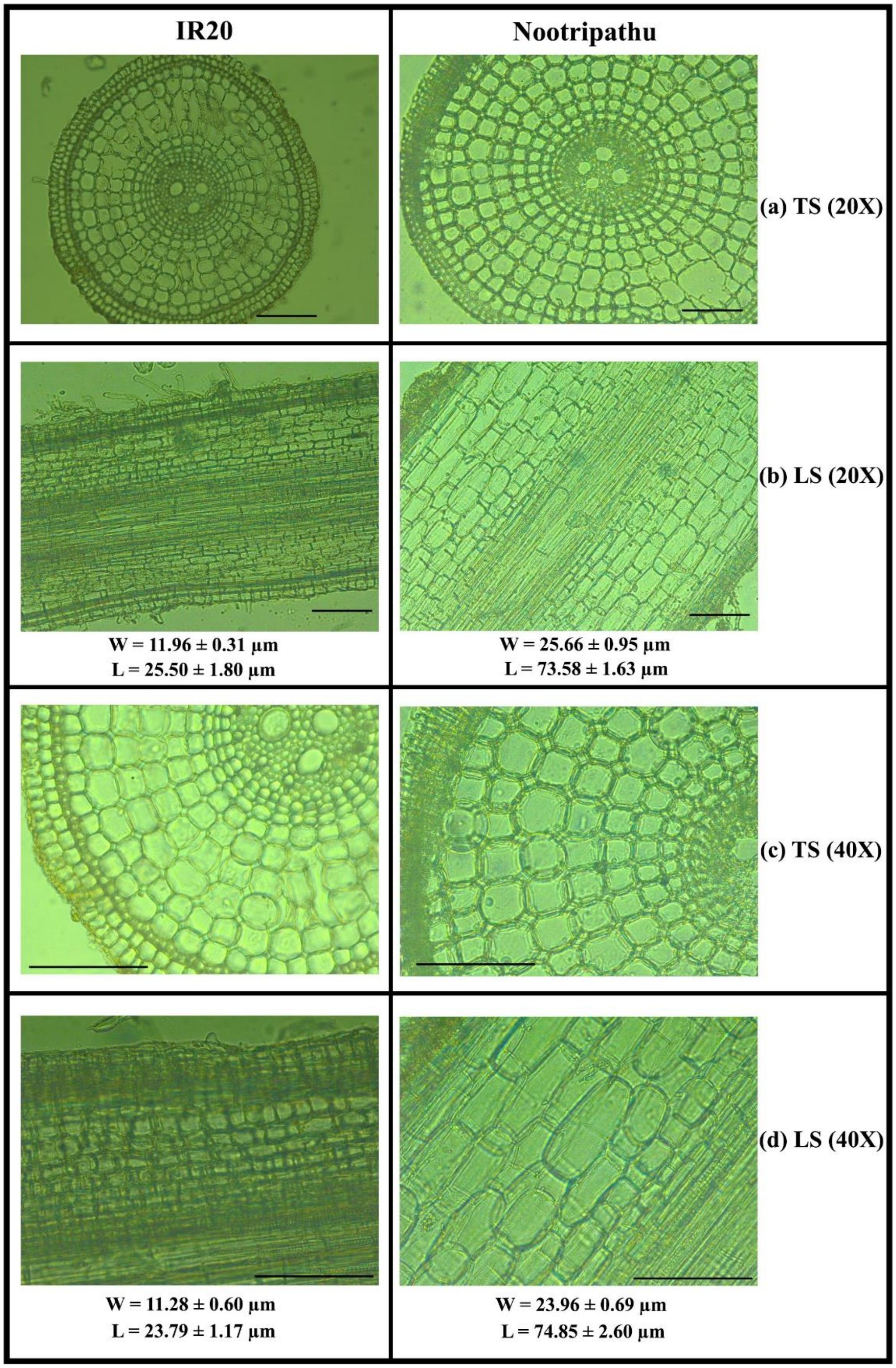

Supplementary Fig. 1. Micrograph of primary roots (4-5 cm from the tip) in 35 days old plants of IR20 and Nootripathu grown under greenhouse conditions; Where W, cell width; L, cell length; TS, transverse section and LS, longitudinal section. Length and width of cells were measured using stage micrometer.

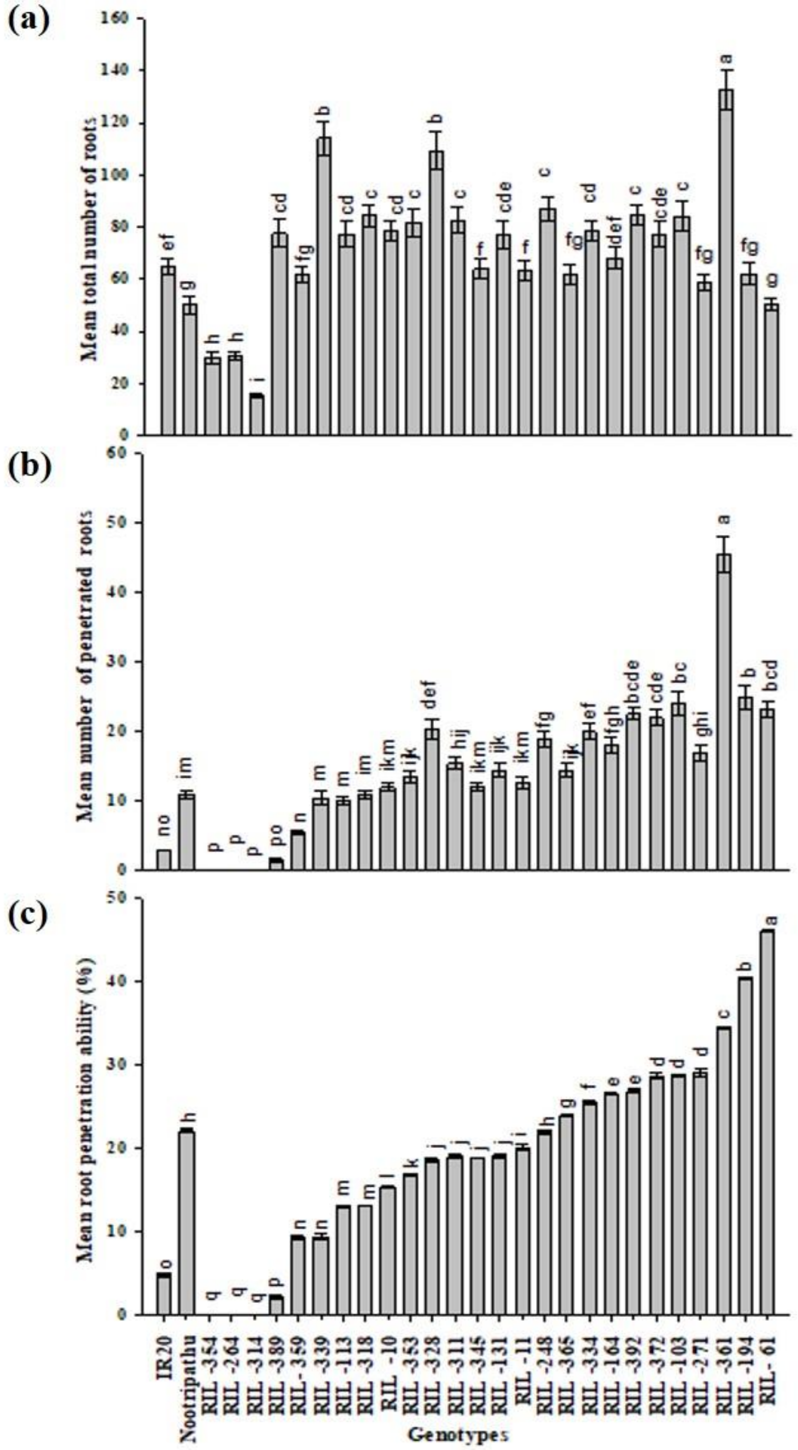

Supplementary Fig. 2. (a) Total number of roots, **(b)** number of penetrated roots and **(c)** root penetration ability (%) of IR20, Nootripathu and their RILs. Data represents the mean of three replications; Vertical bars represent standard error. One-way ANOVA was performed and mean was compared using Fisher's Least Significant Difference (LSD). Genotypes with same alphabets are on-par with each other and different letter alphabets are significantly different ($p<0.01$).

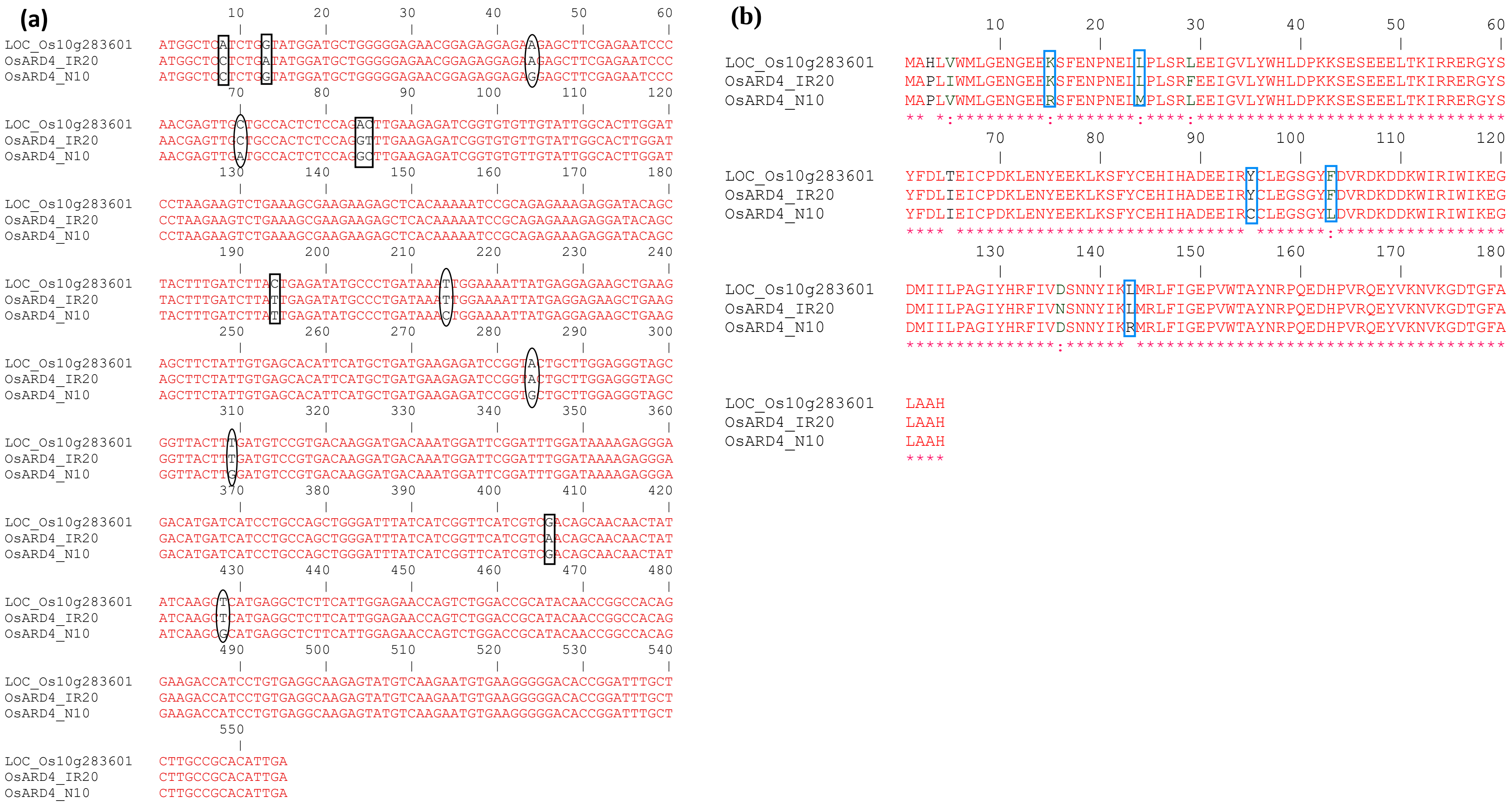

Supplementary Fig. 3. Multiple sequence alignment of nucleotide **(a)** and amino acid **(b)** sequences of *OsARD4* from IR20 (*OsARD4*_IR20); Nootripathu (*OsARD4*_N10) and Nipponbare (LOC_Os10g283601). Circle shows the sequence variation(s) specific to Nootripathu and other variations are shown in rectangular boxes. Amino acid variations specific to Nootripathu are indicated by blue rectangular boxes.

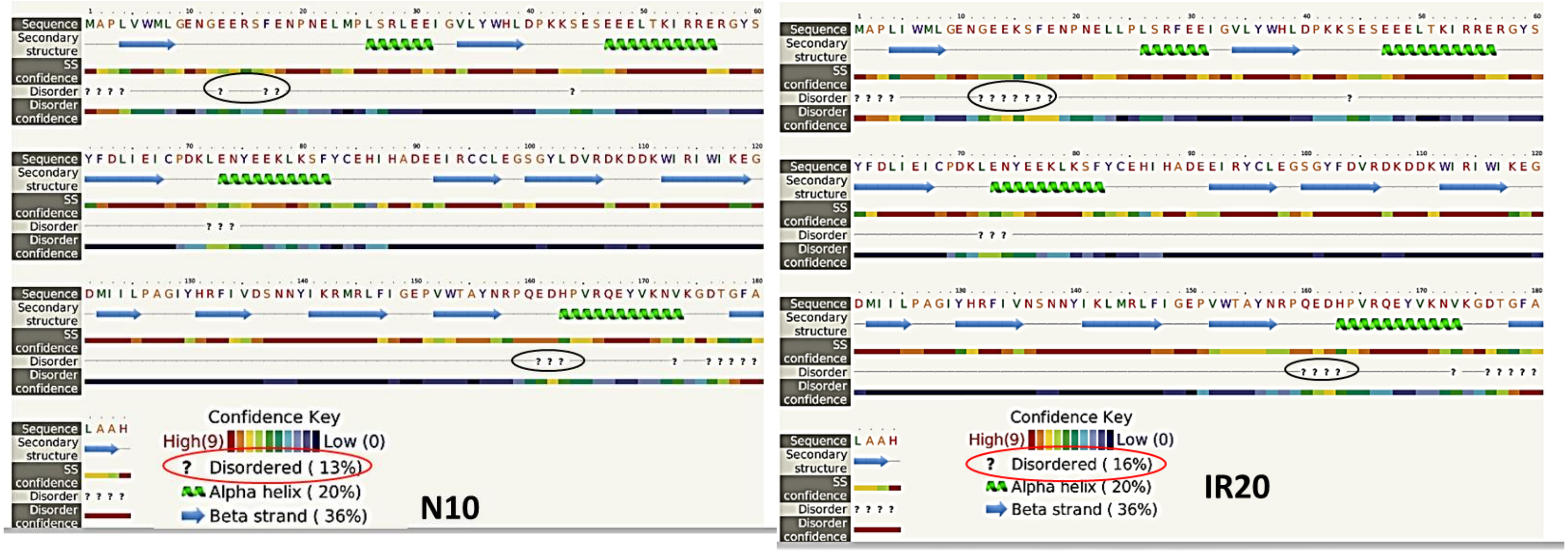

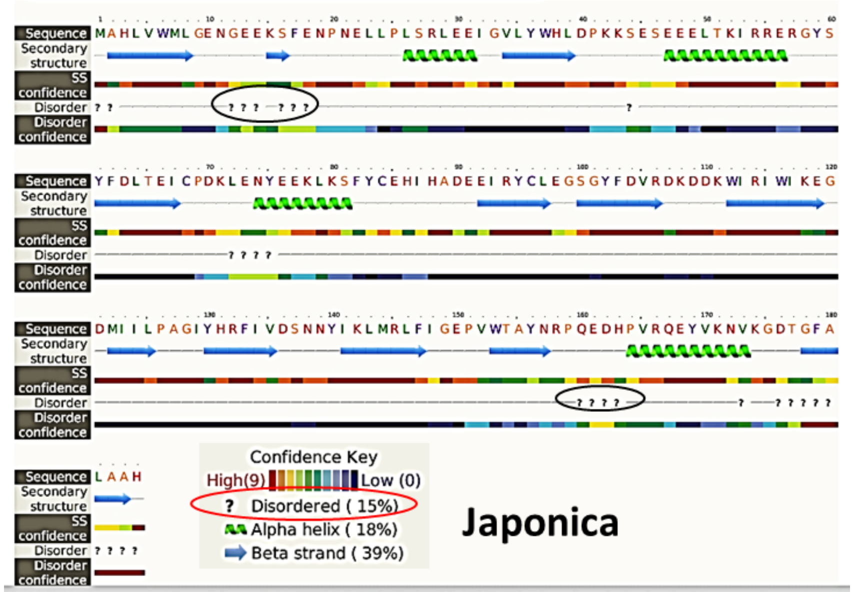

Supplementary Fig. 4. Prediction of secondary structure and disorder among three different alleles of *OsARD4* using Expasy (**P**rotein **H**omology/analog **R**ecognition **E**ngine V 2.0) Phyre 2 software (http://www.sbg.bio.ic.ac.uk/phyre2). Predicted disorders are indicated in red and black circles.

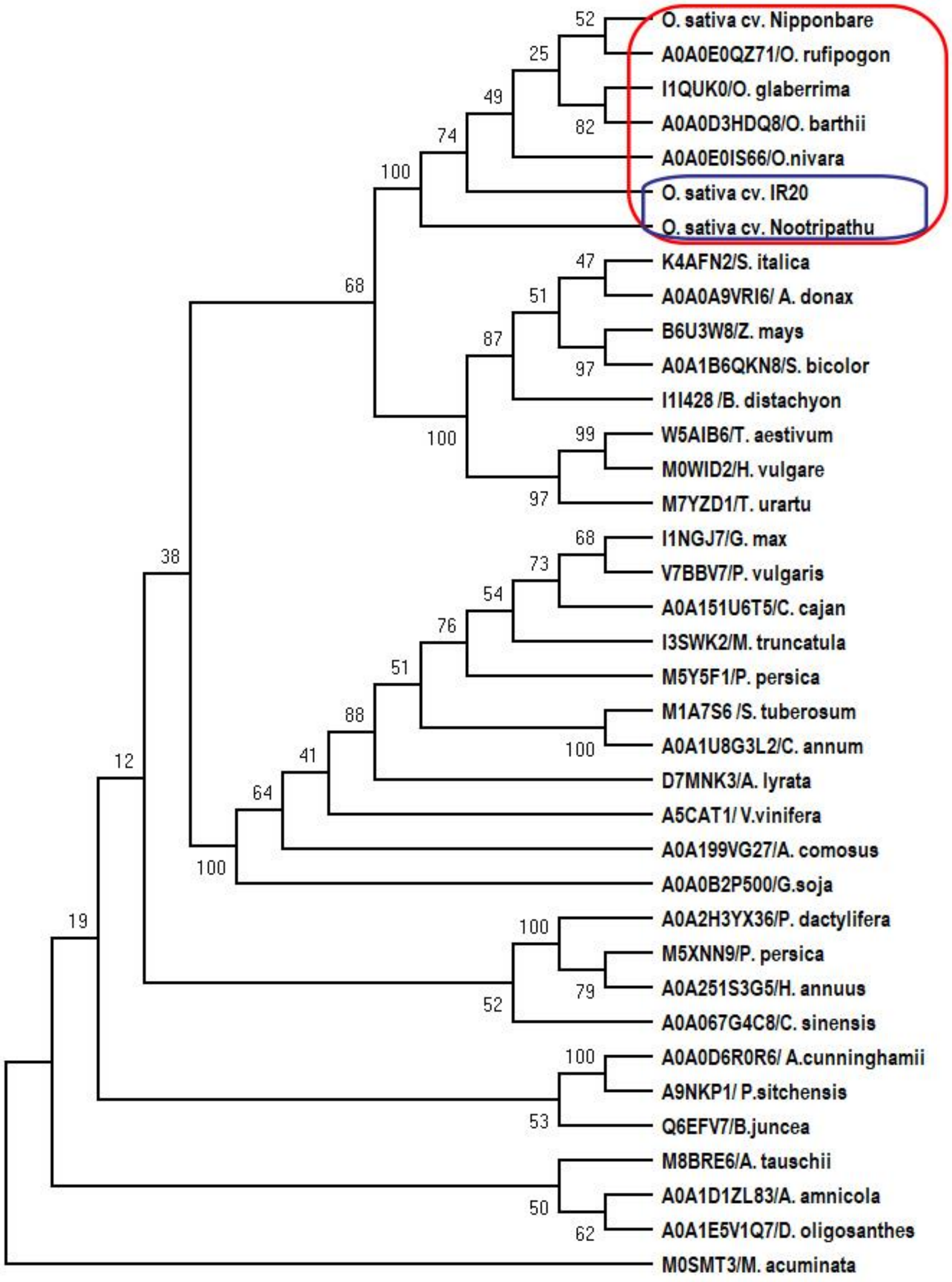

Supplementary Fig. 5. Phylogenetic analysis of *ARD4* sequences from IR20, Nootripathu and Nipponbare along with homologs of other plant species including few members of genus *Oryza* using MEGA 7.0 software. Phylogenetic analysis was carried out using the UPGMA method. The evolutionary distances were computed using the Poisson correction method.

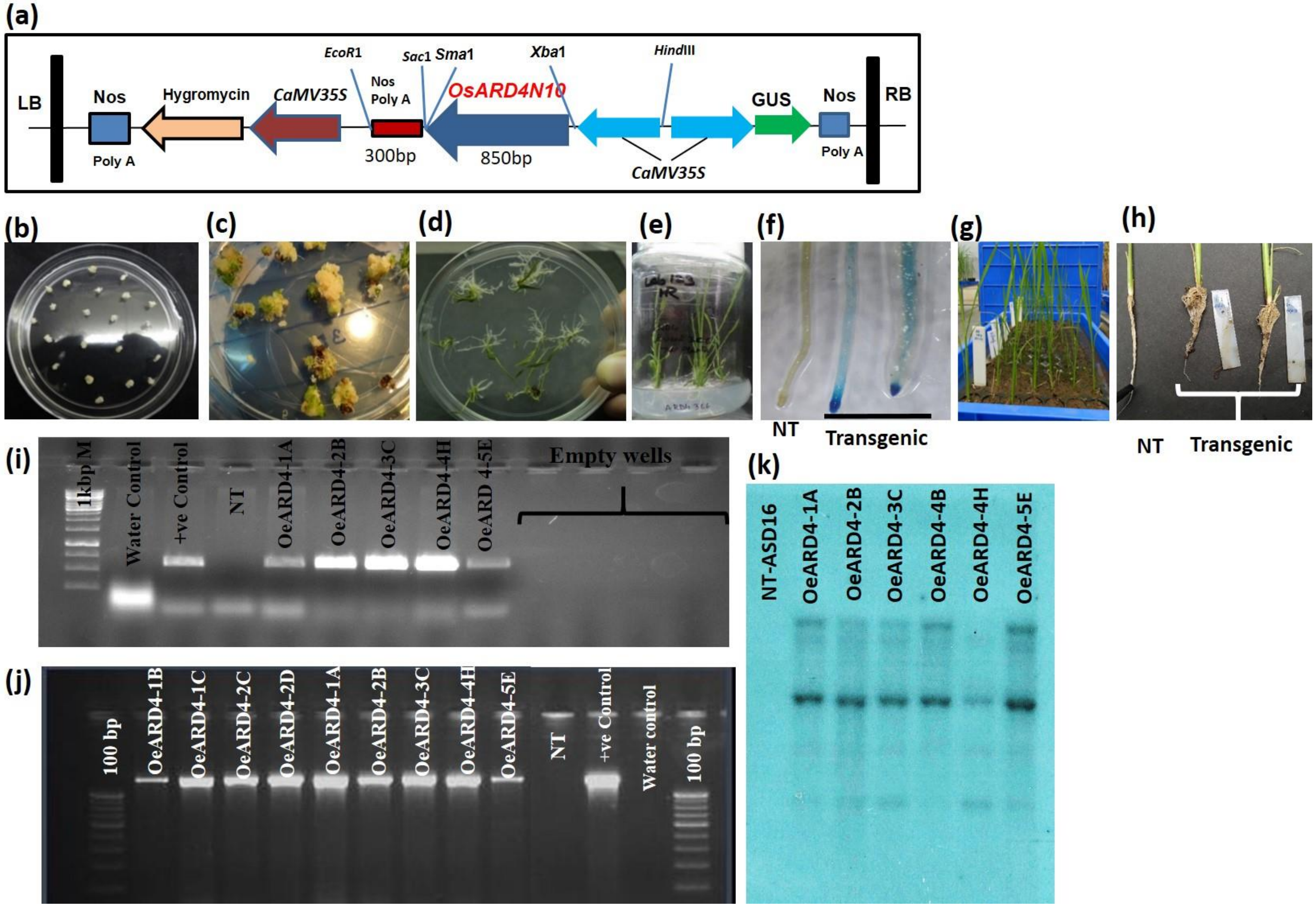

Supplementary Fig. 6 Agrobacterium mediated transformation of rice genotype ASD16 using pCAMBIA 1301 harbouring *OsARD4*$_{\mathbf{Nootripathu}}$. **(a)** pCAMBIA 1301 harboring *OsARD4*$_{\mathrm{Nootripathu}}$ driven by *CaMV35S* promoter; (**b-e)** steps involved in development of transgenic ASD16 plants engineered with *OsARD4*; **(f)** Histochemical GUS staining in roots of NT-ASD16 and transgenic lines in T_0 generation; **(g)** hardening of T_0 plants in portrays under greenhouse conditions**;** **(h)** Roots of NT-ASD16 and transgenic lines before transplanting to pots; **(i-j)** PCR screening of putative transformants using primers specific to GUS gene **(i)** and CaMV35S promoter (forward primer) and hygromycin marker (reverse primer) **(j)**; **(k)** Southern bolt analysis in *OsARD4* transgenic events.

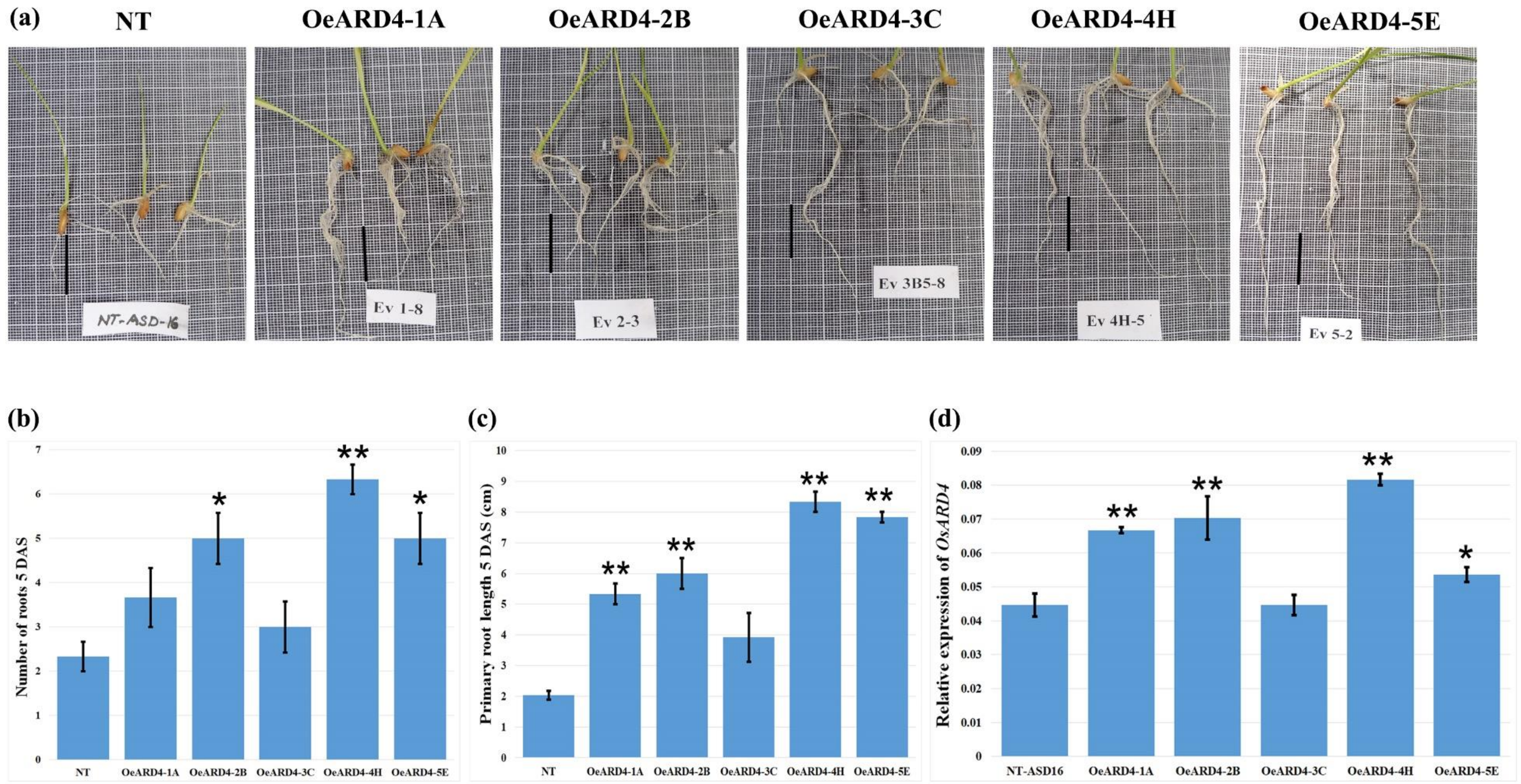

Supplementary Fig. 7. Evaluation of transgenic ASD16 (T_1) for root characteristics and over-expression of OsARD4. (a) Root growth pattern in 5 days old seedlings (grown in petri plates) of non-transgenic ASD16 and putative transgenic ASD16 lines (Scale bar = 2 cm); **(b)** Number of roots in 5 days old seedlings of non-transgenic and transgenic ASD16 plants; **(c)** Length of primary roots in 5 days old seedlings of non-transgenic and transgenic ASD16 plants; **(d)** qRT-PCR analysis of *OsARD4* transcripts in root tissues of non-transgenic and transgenic ASD16 plants. Each data is a mean of three replications. *indicates statistical significance at P< 0.05 and **indicates statistical significance at P< 0.01.

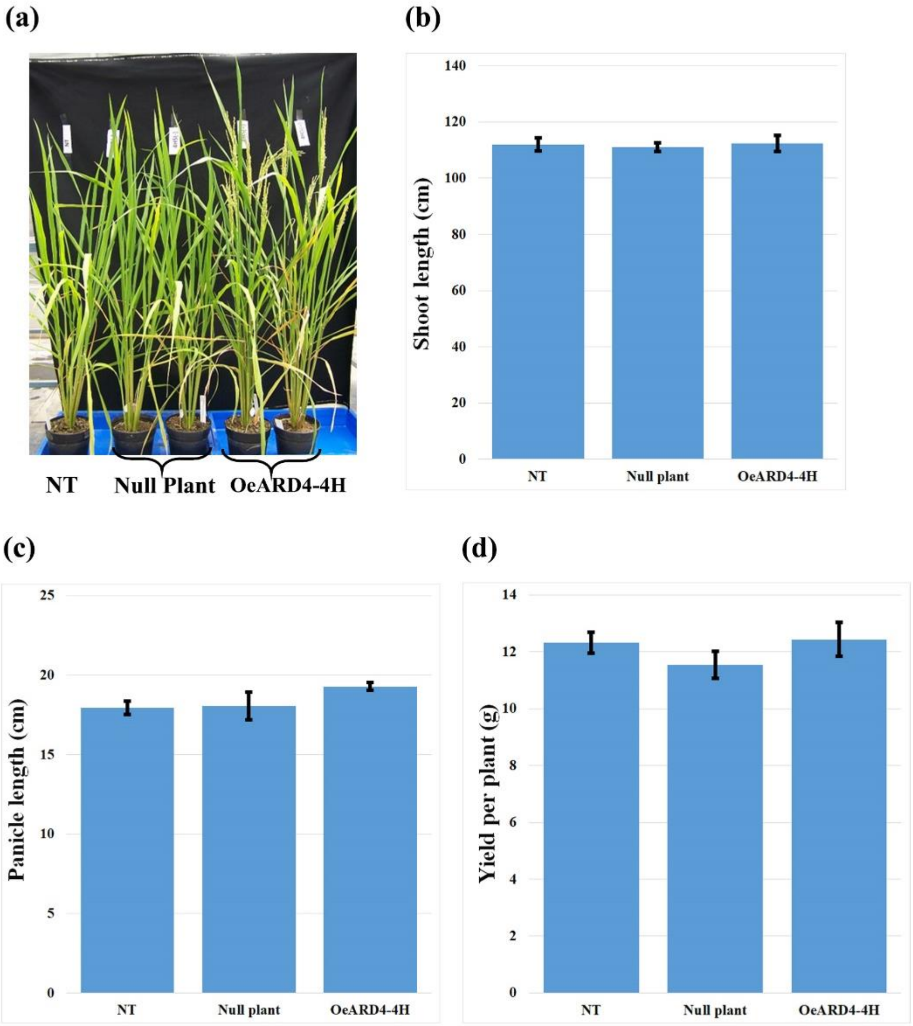

Supplementary Fig. 8. Shoot growth characteristics of OeARD4-4H in comparison with null-plants and non-transgenic ASD16 at maturity stage. (a) General appearance of transgenic ASD16 plants (OeARD4-4H) in comparison with null/non-transgenic plants of ASD16; (b) Shoot length at maturity, (c) panicle length and (d) single plant yield of OeADR4-4H in comparison null(s) and non-transgenic ASD16 plants. Each value is a mean of three replications.

Supplementary Table S1. Observations on anatomical characters in root tips of IR 20 and Nootripathu

Genotypes	Number of cells in divisional zone (100µm scale)	Number of cells in elongation zone (100µm scale)	Length of cells in elongation zone (µm)	Width of cells in elongation zone (µm)	Root cap length (µm)	Number of cells per root cap	Root thickness (µm)
IR20	385[b]	188.75[b]	30.1[b]	20.2[b]	215.0[a]	173.7[b]	265[b]
Nootripathu	502.5[a]	207.5[a]	38.0[a]	23.4[a]	177.5[b]	291.3[a]	390[a]

*Measurements were made in roots of 5 days old seedlings; Each observation is an average of 6 independent measurements.

Supplementary Table 2. Co-localization analysis of DE Proteins against QTLs for root growth related traits in rice

LOCs	Spot ID	Protein Name	Trait name	Chromo some	Left locus	Start Position	Right locus	Stop Position	Reference
LOC_Os03g27450	D1	Putative ADP-ribosylation factor	Maximum root length	3	A123616	15335608	RZ474	25128864	MacMillan et al (2006)
LOC_Os03g27450	D1	Putative ADP-ribosylation factor	Root penetration index	3	A123616	15335608	RZ474	25128864	Price et al (2000)
LOC_Os03g27450	D1	Putative ADP-ribosylation factor	Root fresh weight	3	RM60	105852	C814B	17113562	Li et al (2005)
LOC_Os03g27450	D1	Putative ADP-ribosylation factor	Root fresh weight	3	RM60	105852	C814B	17113562	Li et al (2005)
LOC_Os03g27450	D1	Putative ADP-ribosylation factor	Lateral root length	3	RG409A	3495798	T17	24086309	Zheng et al (2003)
LOC_Os03g27450	D1	Putative ADP-ribosylation factor	Deep root weight	3	PC20M11	5729669	PC20M12	27128592	Kamoshita et al (2002b)
LOC_Os03g27450	D1	Putative ADP-ribosylation factor	Deep root ratio	3	PC20M11	5729669	PC20M12	27128592	Kamoshita et al (2002b)
LOC_Os03g27450	D1	Putative ADP-ribosylation factor	Penetrated root number	3	PC73M13	5729669	PC3M5	27128592	Ali et al (2000)
LOC_Os03g27450	D1	Putative ADP-ribosylation factor	Penetrated root number	3	PC73M13	5729669	PC3M5	27128592	Ali et al (2000)
LOC_Os03g27450	D1	Putative ADP-ribosylation factor	Root penetration index	3	PC73M13	5729669	PC3M5	27128592	Ali et al (2000)
LOC_Os03g27450	D1	Putative ADP-ribosylation factor	Root penetration index	3	PC73M13	5729669	PC3M5	27128592	Ali et al (2000)
LOC_Os03g27450	D1	Putative ADP-ribosylation factor	Root to shoot ratio	3	RM231	2453085	G51	28528927	Li et al (2005)
LOC_Os11g11730	D2	Cell wall adhesion	Maximum root length	11	R2918	2449321	C794	3035893	Xu et al (2004)
LOC_Os11g11730	D2	Cell wall adhesion	Deep root weight per tiller	11	C477	5460751	EM17_10	6686558	Kamoshita et al (2002a)
LOC_Os11g11730	D2	Cell wall adhesion	Root growth rate in depth	11	RM332	2840213	RM167	4073313	Yue et al (2006)
LOC_Os11g11730	D2	Cell wall adhesion	Root dry weight	11	C794	3033385	RG118	4414504	Xu et al (2004)
LOC_Os11g11730	D2	Cell wall adhesion	Root dry weight	11	C794	3033385	RG118	4414504	Xu et al (2004)
LOC_Os11g11730	D2	Cell wall adhesion	Deep root weight	11	R642	2015713	RZ141	4078514	MacMillan et al (2006)
LOC_Os11g11730	D2	Cell wall adhesion	Maximum root length	11	R642	2015713	RZ141	4078514	Price et al (1999)
LOC_Os11g11730	D2	Cell wall adhesion	Deep root ratio	11	RM286	383711	RM332	2840362	Yue et al (2006)
LOC_Os11g11730	D2	Cell wall adhesion	Deep root ratio	11	RM286	383711	RM332	2840362	Yue et al

									(2006)
LOC_Os11g11730	D2	Cell wall adhesion	Maximum root length	11	RM286	383711	RM332	2840362	Yue et al (2006)
LOC_Os11g11730	D2	Cell wall adhesion	Root thickness	11	OSR1	201993	RM202	9001898	Li et al (2005)
LOC_Os01g54700	D3	Retrotransposon protein	Root dry weight	1	RZ19	30870169	RG690	32311314	Yadav et al (1997)
LOC_Os01g54700	D3	Retrotransposon protein	Root thickness	1	RZ19	30870169	RG690	32311314	Yadav et al (1997)
LOC_Os01g54700	D3	Retrotransposon protein	Deep root weight	1	R2417	31427184	RM212	33053654	Kamoshita et al (2002a)
LOC_Os01g54700	D3	Retrotransposon protein	Deep root weight per tiller	1	R2417	31427184	RM212	33053654	Kamoshita et al (2002a)
LOC_Os01g54700	D3	Retrotransposon protein	Maximum root length	1	R2417	31427184	RM212	33053654	Price et al (1999)
LOC_Os01g54700	D3	Retrotransposon protein	Root thickness	1	R2417	31427184	RM212	33053654	Price et al (2002c)
LOC_Os01g54700	D3	Retrotransposon protein	Deep root ratio	1	RG957	31227089	C813	33958675	Kamoshita et al (2002a)
LOC_Os01g54700	D3	Retrotransposon protein	Deep root ratio	1	RG957	31227089	C813	33958675	Kamoshita et al (2002a)
LOC_Os01g54700	D3	Retrotransposon protein	Root thickness	1	ME10_14	31367453	CDO345	37887562	Kamoshita et al (2002a)
LOC_Os01g54700	D3	Retrotransposon protein	Root fresh weight	1	C955	7476866	C813	33958675	Li et al (2005)
LOC_Os01g54700	D3	Retrotransposon protein	Root thickness	1	C904	13902370	C742	40919568	Li et al (2005)
LOC_Os06g47150	D6	Auxin response factor	Root dry weight	6	CDO544	26409713	RG653	29028429	Yadav et al (1997)
LOC_Os06g47150	D6	Auxin response factor	Maximum root length	6	P0547F09	28370801	OSJNBA0069C14	31082396	MacMillan et al (2006)
LOC_Os06g47150	D6	Auxin response factor	Root thickness	6	P0547F09	28370801	OSJNBA0069C14	31082396	MacMillan et al (2006)
LOC_Os06g47150	D6	Auxin response factor	Maximum root length	6	P0547F09	28370801	OSJNBA0069C14	31082396	Price et al (2002c)
LOC_Os06g47150	D6	Auxin response factor	Root branching index	6	RM454	23380386	RM412	30328051	Horii et al (2006)
LOC_Os01g56790	D7	Expressed protein	Deep root weight	1	R2417	31427184	RM212	33053654	Kamoshita et al (2002a)
LOC_Os01g56790	D7	Expressed protein	Deep root weight per tiller	1	R2417	31427184	RM212	33053654	Kamoshita et al (2002a)
LOC_Os01g56790	D7	Expressed protein	Maximum root length	1	R2417	31427184	RM212	33053654	Price et al (1999)

LOC_Os01g56790	D7	Expressed protein	Root thickness	1	R2417	31427184	RM212	33053654	Price et al (2002c)
LOC_Os01g56790	D7	Expressed protein	Maximum root length	1	RG690	32310761	RZ730	34940769	Courtois et al (2003)
LOC_Os01g56790	D7	Expressed protein	Deep root weight per tiller	1	RG690	32310761	RZ730	34940769	Courtois et al (2003)
LOC_Os01g56790	D7	Expressed protein	Deep root to shoot ratio	1	RG690	32310761	RZ730	34940769	Yadav et al (1997)
LOC_Os01g56790	D7	Expressed protein	Deep root ratio	1	RG957	31227089	C813	33958675	Kamoshita et al (2002a)
LOC_Os01g56790	D7	Expressed protein	Deep root ratio	1	RG957	31227089	C813	33958675	Kamoshita et al (2002a)
LOC_Os01g56790	D7	Expressed protein	Root thickness	1	ME10_14	31367453	CDO345	37887562	Kamoshita et al (2002a)
LOC_Os01g56790	D7	Expressed protein	Root fresh weight	1	C955	7476866	C813	33958675	Li et al (2005)
LOC_Os01g56790	D7	Expressed protein	Root thickness	1	C904	13902370	C742	40919568	Li et al (2005)
LOC_Os02g03100	D8	Oxidoreductase, aldo/keto reductase family protein	Penetrated root length	2	PC33M9	744663	PC79M6	5263536	Ali et al (2000)
LOC_Os02g03100	D8	Oxidoreductase, aldo/keto reductase family protein	Penetrated root thickness	2	R1483	744663	RG437	6916662	Zhang et al (2001a)
LOC_Os02g03100	D8	Oxidoreductase, aldo/keto reductase family protein	Root dry weight	2	R1483	744663	RG437	6916662	Zhang et al (2001a)
LOC_Os04g16734	D9	Maturase K	Root dry weight in the 30-60cm layer	4	RM261	6574396	RZ69	11234543	Courtois et al (2003)
LOC_Os04g16734	D9	Maturase K	Deep root to shoot ratio	4	RM261	6574396	RZ69	11234543	Courtois et al (2003)
LOC_Os04g16734	D9	Maturase K	Root number	4	RG190	8610617	RG908	13635003	Hemamalini et al (2000)
LOC_Os04g16734	D9	Maturase K	Root dry weight	4	C820	6898105	C933	12642399	Xu et al (2004)
LOC_Os04g16734	D9	Maturase K	Root to shoot ratio	4	C820	6898105	C933	12642399	Xu et al (2004)
LOC_Os04g16734	D9	Maturase K	Deep root weight per tiller	4	PC11M12	3571596	PC28M1	11021724	Kamoshita et al (2002b)
LOC_Os04g16734	D9	Maturase K	Maximum root length	4	RG190	8610617	C734A	16678378	Price et al (2002c)
LOC_Os04g16734	D9	Maturase K	Deep root ratio	4	RM335	688353	RM307	13141966	Yue et al (2006)
LOC_Os04g16734	D9	Maturase K	Maximum root length	4	RM335	688353	RM307	13141966	Yue et al (2006)
LOC_Os04g16824	D10	Chloroplast 30S ribosomal protein S3	Root dry weight in the 30-60cm layer	4	RM261	6574396	RZ69	11234543	Courtois et al (2003)

LOC_Os04g16824	D10	Chloroplast 30S ribosomal protein S3	Deep root to shoot ratio	4	RM261	6574396	RZ69	11234543	Courtois et al (2003)
LOC_Os04g16824	D10	Chloroplast 30S ribosomal protein S3	Root number	4	RG190	8610617	RG908	13635003	Hemamalini et al (2000)
LOC_Os04g16824	D10	Chloroplast 30S ribosomal protein S3	Root dry weight	4	C820	6898105	C933	12642399	Xu et al (2004)
LOC_Os04g16824	D10	Chloroplast 30S ribosomal protein S3	Root to shoot ratio	4	C820	6898105	C933	12642399	Xu et al (2004)
LOC_Os04g16824	D10	Chloroplast 30S ribosomal protein S3	Deep root weight per tiller	4	PC11M12	3571596	PC28M1	11021724	Kamoshita et al (2002b)
LOC_Os04g16824	D10	Chloroplast 30S ribosomal protein S3	Maximum root length	4	RG190	8610617	C734A	16678378	Price et al (2002c)
LOC_Os04g16824	D10	Chloroplast 30S ribosomal protein S3	Deep root ratio	4	RM335	688353	RM307	13141966	Yue et al (2006)
LOC_Os04g16824	D10	Chloroplast 30S ribosomal protein S3	Maximum root length	4	RM335	688353	RM307	13141966	Yue et al (2006)
LOC_Os01g08510	D11	MAR-binding filament-like protein 1	Deep root ratio	1	RG400	4058510	R1194	4754502	Kamoshita et al (2002a)
LOC_Os01g08510	D11	MAR-binding filament-like protein 1	Deep root weight	1	RG400	4058510	RG532	5763641	MacMillan et al (2006)
LOC_Os01g08510	D11	MAR-binding filament-like protein 1	Root number	1	RG246	3509004	K5	5763641	Zheng et al (2000)
LOC_Os01g08510	D11	MAR-binding filament-like protein 1	Deep root ratio	1	RM428	2606364	RM490	6676652	Yue et al (2006)
LOC_Os01g08510	D11	MAR-binding filament-like protein 1	Root dry weight	1	RM7383	3483439	RM243	7970839	Horii et al (2006)
LOC_Os01g08510	D11	MAR-binding filament-like protein 1	Maximum root length	1	RM7383	3483439	RM23	10704715	Horii et al (2006)
LOC_Os05g10830	D12	Proteophosphoglycan ppg4	Root thickness	5	R2232	4107103	R569	6700408	Price et al (2002c)
LOC_Os05g10830	D12	Proteophosphoglycan ppg4	Lateral root length	5	RG313	2781980	E1328	6944607	Zheng et al (2003)
LOC_Os05g10830	D12	Proteophosphoglycan ppg4	Deep root ratio	5	ME5_13	2781980	G387	12454753	Kamoshita et al (2002a)
LOC_Os05g10830	D12	Proteophosphoglycan ppg4	Deep root weight per tiller	5	ME5_13	2781980	G387	12454753	Kamoshita et al (2002a)
LOC_Os05g10830	D12	Proteophosphoglycan ppg4	Root volume	5	RZ556A	2091558	RG403	12454753	Hemamalini et al (2000)
LOC_Os05g10830	D12	Proteophosphoglycan ppg4	Deep root to shoot ratio	5	RZ556A	2091558	RG403	12454753	Yadav et al (1997)
LOC_Os11g12040	D13	Disease resistance protein RPM1	Maximum root length	11	R2918	2449321	C794	3035893	Xu et al (2004)
LOC_Os11g12040	D13	Disease resistance protein RPM1	Deep root weight per tiller	11	C477	5460751	EM17_10	6686558	Kamoshita et

									al (2002a)
LOC_Os11g12040	D13	Disease resistance protein RPM1	Root growth rate in depth	11	RM332	2840213	RM167	4073313	Yue et al (2006)
LOC_Os11g12040	D13	Disease resistance protein RPM1	Root dry weight	11	C794	3033385	RG118	4414504	Xu et al (2004)
LOC_Os11g12040	D13	Disease resistance protein RPM1	Root dry weight	11	C794	3033385	RG118	4414504	Xu et al (2004)
LOC_Os11g12040	D13	Disease resistance protein RPM1	Deep root weight	11	R642	2015713	RZ141	4078514	MacMillan et al (2006)
LOC_Os11g12040	D13	Disease resistance protein RPM1	Maximum root length	11	R642	2015713	RZ141	4078514	Price et al (1999)
LOC_Os11g12040	D13	Disease resistance protein RPM1	Deep root ratio	11	RM286	383711	RM332	2840362	Yue et al (2006)
LOC_Os11g12040	D13	Disease resistance protein RPM1	Deep root ratio	11	RM286	383711	RM332	2840362	Yue et al (2006)
LOC_Os11g12040	D13	Disease resistance protein RPM1	Maximum root length	11	RM286	383711	RM332	2840362	Yue et al (2006)
LOC_Os11g12040	D13	Disease resistance protein RPM1	Root thickness	11	OSR1	201993	RM202	9001898	Li et al (2005)
LOC_Os10g36190	D14	PPR repeat domain containing protein	Root dry weight in the 60-90cm layer	10	RM258	18014265	RG134	19823295	Courtois et al (2003)
LOC_Os02g37420	D15	Ras-related protein	Maximum root length	2	RM3762	22456471	RM526	26665181	Yue et al (2006)
LOC_Os02g37420	D15	Ras-related protein	Root thickness	2	RG157	19865083	RZ318	24566168	Hemamalini et al (2000)
LOC_Os02g37420	D15	Ras-related protein	Root thickness	2	RG157	19865083	RZ318	24566168	Hemamalini et al (2000)
LOC_Os02g37420	D15	Ras-related protein	Penetrated root number	2	RG171	17484665	G45	22596168	Price et al (2000)
LOC_Os02g37420	D15	Ras-related protein	Root thickness	2	A18438	22595831	G39	27034665	MacMillan et al (2006)
LOC_Os02g37420	D15	Ras-related protein	Root thickness	2	A18438	22595831	G39	27034665	Price et al (2002c)
LOC_Os02g37420	D15	Ras-related protein	Penetrated root number	2	C499	20878430	PC11M1	31106875	Ali et al (2000)
LOC_Os02g37420	D15	Ras-related protein	Root penetration index	2	C499	20878430	PC11M1	31106875	Ali et al (2000)
LOC_Os02g37420	D15	Ras-related protein	Penetrated root number	2	PC33M8	20878430	PC21M1	31106875	Ali et al (2000)
LOC_Os02g37420	D15	Ras-related protein	Penetrated root number	2	PC33M8	20878430	PC21M1	31106875	Ali et al (2000)
LOC_Os02g37420	D15	Ras-related protein	Root penetration index	2	PC33M8	20878430	PC21M1	31106875	Ali et al (2000)

LOC_Os02g37420	D15	Ras-related protein	Root penetration index	2	PC33M8	20878430	PC21M1	31106875	Ali et al (2000)
LOC_Os02g37420	D15	Ras-related protein	Penetrated root number	2	AA7_2B	20878430	AA7_2A	33850388	Ali et al (2000)
LOC_Os02g37420	D15	Ras-related protein	Root penetration index	2	AA7_2B	20878430	AA7_2A	33850388	Ali et al (2000)
LOC_Os02g37420	D15	Ras-related protein	Root fresh weight	2	G1327	4524663	RM263	25865568	Li et al (2005)
LOC_Os07g09890	U1	Hexokinase	Deep root weight	7	RZ488	4573513	RG477	6779821	Courtois et al (2003)
LOC_Os07g09890	U1	Hexokinase	Root number	7	PC75M8	2678661	PC32M1	17820931	Ali et al (2000)
LOC_Os08g02050	U2	Protein kinase family protein	Root thickness	8	RM337	152299	P0498H04	729015	Price et al (1999)
LOC_Os08g02050	U2	Protein kinase family protein	Root thickness	8	PC27M15	460381	C1121	5327118	Kamoshita et al (2002b)
LOC_Os10g28360	U3	1,2-dihydroxy-3-keto-5-methylthiopentene dioxygenase	Maximum root length	10	C1633	14607047	C677	16410869	Xu et al (2004)
LOC_Os10g28360	U3	1,2-dihydroxy-3-keto-5-methylthiopentene dioxygenase	Root penetration index	10	RZ892A	12902744	BCD386	19023681	Ali et al (2000)
LOC_Os10g28360	U3	1,2-dihydroxy-3-keto-5-methylthiopentene dioxygenase	Penetrated root thickness	10	RZ892A	12902744	BCD386	19023681	Ali et al (2000)
LOC_Os07g44430	U4	Peroxiredoxin	Maximum root length	7	RM234	25472688	C507	26705745	Price et al (1999)
LOC_Os07g44430	U4	Peroxiredoxin	Deep root weight per tiller	7	CDO497	25103215	CDO418	26916502	Yadav et al (1997)
LOC_Os07g44430	U4	Peroxiredoxin	Maximum root length	7	CDO497	25103215	CDO418	26916502	Yadav et al (1997)
LOC_Os07g44430	U4	Peroxiredoxin	Root dry weight in the 00-30cm layer	7	RG146B	25989310	RM248	29340115	Courtois et al (2003)
LOC_Os07g44430	U4	Peroxiredoxin	Root thickness	7	RM47	25806439	RM172	29561451	Li et al (2005)
LOC_Os07g44430	U4	Peroxiredoxin	Root dry weight	7	RM47	25806439	RM172	29561451	Li et al (2005)
LOC_Os07g44430	U4	Peroxiredoxin	Maximum root length	7	ME7_1	23178645	EM18_4	28792010	Kamoshita et al (2002a)
LOC_Os07g46550	U6	Annexin	Root penetration index	7	CDO418	26912502	RZ978	28411821	Zheng et al (2000)
LOC_Os07g46550	U6	Annexin	Root dry weight	7	CDO418	26912502	RZ978	28411821	Yadav et al (1997)
LOC_Os07g46550	U6	Annexin	Deep root weight	7	CDO418	26912502	RZ978	28411821	Yadav et al (1997)
LOC_Os07g46550	U6	Annexin	Deep root to shoot ratio	7	CDO418	26912502	RZ978	28411821	Yadav et al

									(1997)
LOC_Os07g46550	U6	Annexin	Deep root ratio	7	RM134	26636534	RM248	29340115	Yue et al (2006)
LOC_Os07g46550	U6	Annexin	Root growth rate in volume	7	RM134	26636534	RM248	29340115	Yue et al (2006)
LOC_Os07g46550	U6	Annexin	Maximum root length	7	A123714	26704922	A123612	29467498	Price et al (2002c)
LOC_Os07g46550	U6	Annexin	Root dry weight in the 00-30cm layer	7	RG146B	25989310	RM248	29340115	Courtois et al (2003)
LOC_Os07g46550	U6	Annexin	Root thickness	7	RM47	25806439	RM172	29561451	Li et al (2005)
LOC_Os07g46550	U6	Annexin	Root dry weight	7	RM47	25806439	RM172	29561451	Li et al (2005)
LOC_Os07g46550	U6	Annexin	Maximum root length	7	ME7_1	23178645	EM18_4	28792010	Kamoshita et al (2002a)
LOC_Os08g40090	U7	Leucine rich repeat containing protein	Deep root number	8	R202	24185548	RG598	27735535	Price et al (2002c)
LOC_Os08g40090	U7	Leucine rich repeat containing protein	Maximum root length	8	R202	24185548	RG598	27735535	Price et al (2002c)
LOC_Os08g40090	U7	Leucine rich repeat containing protein	Root thickness	8	RZ997A	22925855	RZ572	27555414	Nguyen et al (2004)
LOC_Os04g24610	U9	Expressed protein	Maximum root length	4	RM307	13141838	RM471	18824943	Yue et al (2006)
LOC_Os04g24610	U9	Expressed protein	Root thickness	4	RZ69	11234426	RG449	17696172	Courtois et al (2003)
LOC_Os04g24610	U9	Expressed protein	Deep root weight	4	RZ69	11234426	RG449	17696172	Courtois et al (2003)
LOC_Os04g24610	U9	Expressed protein	Deep root weight per tiller	4	RZ69	11234426	RG449	17696172	Courtois et al (2003)
LOC_Os04g24610	U9	Expressed protein	Maximum root length	4	RG190	8610617	C734A	16678378	Price et al (2002c)
LOC_Os04g24610	U9	Expressed protein	Root penetration index	4	EM14_5	11234426	ME2_13	21453222	Nguyen et al (2004)
LOC_Os04g24610	U9	Expressed protein	Root penetration index	4	RZ69	11234426	RZ565	21453222	Zhang et al (2001a)
LOC_Os03g18850	U10	Pathogenesis-related Bet v I family protein	Root penetration index	3	RG369	8964495	R2170	14291501	Zhang et al (2001a)
LOC_Os03g18850	U10	Pathogenesis-related Bet v I family protein	Root pulling force	3	RG369	8964495	R2170	14291501	Zhang et al (2001a)
LOC_Os03g18850	U10	Pathogenesis-related Bet v I family protein	Maximum root length	3	RG191	5729669	A12451	12147899	Price et al (2002c)
LOC_Os03g18850	U10	Pathogenesis-related Bet v I family	Root thickness	3	RG191	5729669	A12451	12147899	Price et al

		protein							(2002c)
LOC_Os03g18850	U10	Pathogenesis-related Bet v I family protein	Lateral root number	3	RG191	5729669	AAC_CA G5	12407510	Zheng et al (2003)
LOC_Os03g18850	U10	Pathogenesis-related Bet v I family protein	Root dry weight	3	RZ536C	5729669	RZ284	15472481	Courtois et al (2003)
LOC_Os03g18850	U10	Pathogenesis-related Bet v I family protein	Root fresh weight	3	RM60	105852	C814B	17113562	Li et al (2005)
LOC_Os03g18850	U10	Pathogenesis-related Bet v I family protein	Lateral root length	3	RG409A	3495798	T17	24086309	Zheng et al (2003)
LOC_Os03g18850	U10	Pathogenesis-related Bet v I family protein	Deep root weight	3	PC20M11	5729669	PC20M12	27128592	Kamoshita et al (2002b)
LOC_Os03g18850	U10	Pathogenesis-related Bet v I family protein	Deep root ratio	3	PC20M11	5729669	PC20M12	27128592	Kamoshita et al (2002b)
LOC_Os03g18850	U10	Pathogenesis-related Bet v I family protein	Penetrated root number	3	PC73M13	5729669	PC3M5	27128592	Ali et al (2000)
LOC_Os03g18850	U10	Pathogenesis-related Bet v I family protein	Penetrated root number	3	PC73M13	5729669	PC3M5	27128592	Ali et al (2000)
LOC_Os03g18850	U10	Pathogenesis-related Bet v I family protein	Root penetration index	3	PC73M13	5729669	PC3M5	27128592	Ali et al (2000)
LOC_Os03g18850	U10	Pathogenesis-related Bet v I family protein	Root penetration index	3	PC73M13	5729669	PC3M5	27128592	Ali et al (2000)
LOC_Os03g18850	U10	Pathogenesis-related Bet v I family protein	Root to shoot ratio	3	RM231	2453085	G51	28528927	Li et al (2005)
LOC_Os08g06060	U11	CGMC_MAPKCMGC_2_ERK.13 - CGMC includes CDA, MAPK, GSK3, and CLKC kinases	Root thickness	8	P0680F05	1996979	R902	4688027	MacMillan et al (2006)
LOC_Os08g06060	U11	CGMC_MAPKCMGC_2_ERK.13 - CGMC includes CDA, MAPK, GSK3, and CLKC kinases	Penetrated root number	8	P0680F05	1996979	R902	4688027	Price et al (2000)
LOC_Os08g06060	U11	CGMC_MAPKCMGC_2_ERK.13 - CGMC includes CDA, MAPK, GSK3, and CLKC kinases	Root penetration index	8	P0680F05	1996979	R902	4688027	Price et al (2000)
LOC_Os08g06060	U11	CGMC_MAPKCMGC_2_ERK.13 - CGMC includes CDA, MAPK, GSK3, and CLKC kinases	Root thickness	8	PC27M15	460381	C1121	5327118	Kamoshita et al (2002b)
LOC_Os05g28530	U12	Glutaredoxin	Root dry weight	5	RG403	12454537	RG13	18875558	Yadav et al (1997)
LOC_Os05g28530	U12	Glutaredoxin	Root pulling force	5	RG403	12454537	RM164	19196736	Zhang et al (2001a)
LOC_Os05g28530	U12	Glutaredoxin	Maximum root length	5	R569	6699817	RG13	18875558	Price et al (1999)

LOC_Os04g50790	U13	RNA recognition motif	Root thickness	4	RZ23	29862697	RM255	30772540	Courtois et al (2003)
LOC_Os04g50790	U13	RNA recognition motif	Root penetration index	4	RG939	27292850	RG476B	31065616	Zhang et al (2001a)
LOC_Os04g50790	U13	RNA recognition motif	Root thickness	4	RG939	27292850	RG476B	31065616	Zhang et al (2001a)
LOC_Os04g50790	U13	RNA recognition motif	Penetrated root thickness	4	RG939	27292850	RG476B	31065616	Zhang et al (2001a)
LOC_Os04g50790	U13	RNA recognition motif	Penetrated root dry weight	4	RG939	27292850	RG476B	31065616	Zhang et al (2001a)
LOC_Os04g50790	U13	RNA recognition motif	Penetrated root thickness	4	RG939	27292850	RZ905	31065616	Nguyen et al (2004)
LOC_Os04g50790	U13	RNA recognition motif	Root thickness	4	RG939	27292850	RZ905	31065616	Nguyen et al (2004)
LOC_Os04g50790	U13	RNA recognition motif	Maximum root length	4	RG163	27292850	RZ590	32449446	Hemamalini et al (2000)
LOC_Os04g50790	U13	RNA recognition motif	Penetrated root thickness	4	RG163	27292850	RZ590	32449446	Zheng et al (2000)
LOC_Os04g50790	U13	RNA recognition motif	Root number	4	RG163	27292850	RM349	32499619	Price et al (2000)
LOC_Os04g50790	U13	RNA recognition motif	Root to shoot ratio	4	RG163	27292850	RM349	32499619	Price et al (2002c)
LOC_Os04g50790	U13	RNA recognition motif	Lateral root number	4	RM252	25178944	AGG_CAG7	34828907	Zheng et al (2003)
LOC_Os12g06560	U14	Expressed protein	Root to shoot ratio	11	Y6854L	26967740	RM224	27202086	Xu et al (2004)
LOC_Os12g06560	U14	Expressed protein	Root thickness	11	G181	26210617	RM224	27202086	Li et al (2005)
LOC_Os12g06560	U14	Expressed protein	Root dry weight	11	G181	26210617	RM224	27202086	Li et al (2005)
LOC_Os12g06560	U14	Expressed protein	Penetrated root length	11	G1465	24200468	C950	26041524	Zhang et al (2001a)
LOC_Os12g06560	U14	Expressed protein	Penetrated root length	11	G1465	24200468	C950	26041524	Nguyen et al (2004)
LOC_Os12g06560	U14	Expressed protein	Root pulling force	11	ME2_6	19172832	RM21	19173094	Nguyen et al (2004)
LOC_Os12g06560	U14	Expressed protein	Root dry weight in the 00-30cm layer	11	RM21	19172832	CDO365	19565672	Courtois et al (2003)
LOC_Os12g06560	U14	Expressed protein	Root dry weight in the 60-90cm layer	11	RM21	19172832	CDO365	19565672	Courtois et al (2003)
LOC_Os12g06560	U14	Expressed protein	Root dry weight	11	RM21	19172832	CDO365	19565672	Courtois et al (2003)
LOC_Os12g06560	U14	Expressed protein	Root thickness	11	C189	21425337	OSJNBA0	22099273	MacMillan et

							074L11		al (2006)
LOC_Os12g06560	U14	Expressed protein	Penetrated root number	11	C189	21425337	OSJNBA0 074L11	22099273	Price et al (2000)
LOC_Os12g06560	U14	Expressed protein	Root penetration index	11	C189	21425337	OSJNBA0 074L11	22099273	Price et al (2000)
LOC_Os12g06560	U14	Expressed protein	Maximum root length	11	C189	21425337	OSJNBA0 074L11	22099273	Price et al (2002c)
LOC_Os12g06560	U14	Expressed protein	Root to total biomass ratio	11	OSJNBA0 074L11	22095414	OSJNBA0 007P22	22840721	MacMillan et al (2006)
LOC_Os12g06560	U14	Expressed protein	Root number	11	OSJNBA0 074L11	22095414	OSJNBA0 007P22	22840721	Price et al (2000)
LOC_Os12g06560	U14	Expressed protein	Root thickness	11	OSJNBA0 074L11	22095414	OSJNBA0 007P22	22840721	Price et al (2002c)
LOC_Os12g06560	U14	Expressed protein	Root to shoot ratio	11	RM229	18407879	RM21	19173094	Li et al (2005)
LOC_Os12g06560	U14	Expressed protein	Deep root ratio	11	PC41M14	17353847	PC48M15	18179510	Kamoshita et al (2002b)
LOC_Os12g06560	U14	Expressed protein	Deep root weight	11	PC48M15	17353847	PC31M8	18179510	Kamoshita et al (2002b)
LOC_Os12g06560	U14	Expressed protein	Penetrated root length	11	PC74M2	19563844	RG103	20337612	Ali et al (2000)
LOC_Os12g06560	U14	Expressed protein	Deep root weight per tiller	11	C477	5460751	EM17_10	6686558	Kamoshita et al (2002a)
LOC_Os12g06560	U14	Expressed protein	Maximum root length	11	G4001	22816378	RM254	23764518	Xu et al (2004)
LOC_Os12g06560	U14	Expressed protein	Maximum root length	11	RM206	22014679	RG1109	23155291	Kamoshita et al (2002a)
LOC_Os12g06560	U14	Expressed protein	Root growth rate in depth	11	RM332	2840213	RM167	4073313	Yue et al (2006)
LOC_Os12g06560	U14	Expressed protein	Root dry weight	11	C794	3033385	RG118	4414504	Xu et al (2004)
LOC_Os12g06560	U14	Expressed protein	Root dry weight	11	C794	3033385	RG118	4414504	Xu et al (2004)
LOC_Os12g06560	U14	Expressed protein	Deep root ratio	11	RM287	16767319	RM229	18408009	Yue et al (2006)
LOC_Os12g06560	U14	Expressed protein	Maximum root length	11	RM287	16767319	RM229	18408009	Yue et al (2006)
LOC_Os12g06560	U14	Expressed protein	Root growth rate in depth	11	RM287	16767319	RM229	18408009	Yue et al (2006)
LOC_Os12g06560	U14	Expressed protein	Root growth rate in depth	11	RM287	16767319	RM229	18408009	Yue et al (2006)
LOC_Os12g06560	U14	Expressed protein	Deep root weight	11	G257	17353847	ME2_6	19173094	Kamoshita et al (2002a)

LOC_Os12g06560	U14	Expressed protein	Deep root weight	11	G257	17353847	ME2_6	19173094	Kamoshita et al (2002a)
LOC_Os12g06560	U14	Expressed protein	Deep root ratio	11	G257	17353847	ME2_6	19173094	Kamoshita et al (2002a)
LOC_Os12g06560	U14	Expressed protein	Root pulling force	11	G257	17353847	RM21	19173094	Zhang et al (2001a)
LOC_Os12g06560	U14	Expressed protein	Deep root weight	11	R642	2015713	RZ141	4078514	MacMillan et al (2006)
LOC_Os12g06560	U14	Expressed protein	Maximum root length	11	R642	2015713	RZ141	4078514	Price et al (1999)
LOC_Os12g06560	U14	Expressed protein	Deep root ratio	11	ME6_7	19563844	ME7_2	22014851	Kamoshita et al (2002a)
LOC_Os12g06560	U14	Expressed protein	Deep root weight per tiller	11	ME7_2	19563844	EM18_19	22014851	Kamoshita et al (2002a)
LOC_Os12g06560	U14	Expressed protein	Root thickness	11	ME7_2	19563844	EM18_19	22014851	Kamoshita et al (2002a)
LOC_Os12g06560	U14	Expressed protein	Root thickness	11	RM229	18407879	C189	21426217	MacMillan et al (2006)
LOC_Os12g06560	U14	Expressed protein	Root thickness	11	RM229	18407879	C189	21426217	Price et al (2002c)
LOC_Os12g06560	U14	Expressed protein	Root growth rate in volume	11	RM202	9001608	RM287	16767617	Yue et al (2006)
LOC_Os12g06560	U14	Expressed protein	Root growth rate in volume	11	RM202	9001608	RM287	16767617	Yue et al (2006)
LOC_Os12g06560	U14	Expressed protein	Root growth rate in volume	11	RM202	9001608	RM287	16767617	Yue et al (2006)
LOC_Os12g06560	U14	Expressed protein	Root growth rate in volume	11	RM202	9001608	RM287	16767617	Yue et al (2006)
LOC_Os12g06560	U14	Expressed protein	Root dry weight	11	RM202	9001608	RM21	19173094	Horii et al (2006)
LOC_Os12g06560	U14	Expressed protein	Root branching index	11	RM202	9001608	RM21	19173094	Horii et al (2006)
LOC_Os12g06560	U14	Expressed protein	Root thickness	11	OSR1	201993	RM202	9001898	Li et al (2005)
LOC_Os12g06560	U14	Expressed protein	Deep root number	12	RM247	3185384	G124	4103357	Price et al (2002c)
LOC_Os01g68630	U15	Leaf senescence related protein	Deep root weight	1	RZ14	39687395	P0678F11	40216906	MacMillan et al (2006)
LOC_Os01g68630	U15	Leaf senescence related protein	Deep root weight	1	RZ14	39687395	P0678F11	40216906	MacMillan et al (2006)
LOC_Os01g68630	U15	Leaf senescence related protein	Maximum root length	1	RZ14	39687395	P0678F11	40216906	MacMillan et al (2006)

LOC_Os01g68630	U15	Leaf senescence related protein	Maximum root length	1	RZ14	39687395	P0678F11	40216906	MacMillan et al (2006)
LOC_Os01g68630	U15	Leaf senescence related protein	Deep root number	1	RZ14	39687395	P0678F11	40216906	Price et al (2002c)
LOC_Os01g68630	U15	Leaf senescence related protein	Maximum root length	1	RZ14	39687395	P0678F11	40216906	Price et al (2002c)
LOC_Os01g68630	U15	Leaf senescence related protein	Root thickness	1	RZ14	39687395	RZ801	40566030	Courtois et al (2003)
LOC_Os01g68630	U15	Leaf senescence related protein	Root dry weight	1	RZ649B	39433049	RZ801	40566030	Courtois et al (2003)
LOC_Os01g68630	U15	Leaf senescence related protein	Root growth rate in volume	1	RM472	37889242	RM104	40167060	Yue et al (2006)
LOC_Os01g68630	U15	Leaf senescence related protein	Root growth rate in volume	1	RM472	37889242	RM104	40167060	Yue et al (2006)
LOC_Os01g68630	U15	Leaf senescence related protein	Root volume	1	RM472	37889242	RM104	40167060	Yue et al (2006)
LOC_Os01g68630	U15	Leaf senescence related protein	Root volume	1	RM472	37889242	RM104	40167060	Yue et al (2006)
LOC_Os01g68630	U15	Leaf senescence related protein	Penetrated root thickness	1	RZ730	34940404	RZ801	40566030	Zheng et al (2000)
LOC_Os01g68630	U15	Leaf senescence related protein	Deep root weight	1	RZ730	34940404	RZ801	40566030	Yadav et al (1997)
LOC_Os01g68630	U15	Leaf senescence related protein	Deep root weight per tiller	1	RZ730	34940404	RZ801	40566030	Yadav et al (1997)
LOC_Os01g68630	U15	Leaf senescence related protein	Root thickness	1	PC31M10	33053493	PC32M5	40700598	Kamoshita et al (2002b)
LOC_Os01g68630	U15	Leaf senescence related protein	Root thickness	1	C904	13902370	C742	40919568	Li et al (2005)
LOC_Os01g68630	U15	Leaf senescence related protein	Penetrated root length	1	PC15M10	40700034	PC17M5	33958675	Ali et al (2000)
LOC_Os01g68630	U15	Leaf senescence related protein	Penetrated root thickness	1	PC3M3	40700034	PC15M11	33958675	Ali et al (2000)
LOC_Os07g31370	U16	Ras-related protein	Maximum root length	7	G20	17526417	C451	20346668	Price et al (1999)
LOC_Os07g31370	U16	Ras-related protein	Root volume	7	RZ337B	17161471	CDO497	25103438	Hemamalini et al (2000)
LOC_Os07g31370	U16	Ras-related protein	Penetrated root number	7	RZ337B	17161471	CDO497	25103438	Zheng et al (2000)
LOC_Os07g31370	U16	Ras-related protein	Root thickness	7	RZ337B	17161471	RM234	25472820	Courtois et al (2003)
LOC_Os07g31370	U16	Ras-related protein	Root number	7	PGMS0_7	6779215	CDO59	20732663	Hemamalini et al (2000)

LOC_Os11g45180	U18	NBS-LRR disease resistance protein	Root to shoot ratio	11	Y6854L	26967740	RM224	27202086	Xu et al (2004)
LOC_Os11g45180	U18	NBS-LRR disease resistance protein	Root thickness	11	G181	26210617	RM224	27202086	Li et al (2005)
LOC_Os11g45180	U18	NBS-LRR disease resistance protein	Root dry weight	11	G181	26210617	RM224	27202086	Li et al (2005)
LOC_Os11g45180	U18	NBS-LRR disease resistance protein	Penetrated root length	11	G1465	24200468	C950	26041524	Zhang et al (2001a)
LOC_Os11g45180	U18	NBS-LRR disease resistance protein	Penetrated root length	11	G1465	24200468	C950	26041524	Nguyen et al (2004)
LOC_Os11g45180	U18	NBS-LRR disease resistance protein	Root pulling force	11	ME2_6	19172832	RM21	19173094	Nguyen et al (2004)
LOC_Os11g45180	U18	NBS-LRR disease resistance protein	Maximum root length	11	R2918	2449321	C794	3035893	Xu et al (2004)
LOC_Os11g45180	U18	NBS-LRR disease resistance protein	Root dry weight in the 00-30cm layer	11	RM21	19172832	CDO365	19565672	Courtois et al (2003)
LOC_Os11g45180	U18	NBS-LRR disease resistance protein	Root dry weight in the 60-90cm layer	11	RM21	19172832	CDO365	19565672	Courtois et al (2003)
LOC_Os11g45180	U18	NBS-LRR disease resistance protein	Root dry weight	11	RM21	19172832	CDO365	19565672	Courtois et al (2003)
LOC_Os11g45180	U18	NBS-LRR disease resistance protein	Root thickness	11	C189	21425337	OSJNBA0 074L11	22099273	MacMillan et al (2006)
LOC_Os11g45180	U18	NBS-LRR disease resistance protein	Penetrated root number	11	C189	21425337	OSJNBA0 074L11	22099273	Price et al (2000)
LOC_Os11g45180	U18	NBS-LRR disease resistance protein	Root penetration index	11	C189	21425337	OSJNBA0 074L11	22099273	Price et al (2000)
LOC_Os11g45180	U18	NBS-LRR disease resistance protein	Maximum root length	11	C189	21425337	OSJNBA0 074L11	22099273	Price et al (2002c)
LOC_Os11g45180	U18	NBS-LRR disease resistance protein	Root to total biomass ratio	11	OSJNBA0 074L11	22095414	OSJNBA0 007P22	22840721	MacMillan et al (2006)
LOC_Os11g45180	U18	NBS-LRR disease resistance protein	Root number	11	OSJNBA0 074L11	22095414	OSJNBA0 007P22	22840721	Price et al (2000)
LOC_Os11g45180	U18	NBS-LRR disease resistance protein	Root thickness	11	OSJNBA0 074L11	22095414	OSJNBA0 007P22	22840721	Price et al (2002c)
LOC_Os11g45180	U18	NBS-LRR disease resistance protein	Root to shoot ratio	11	RM229	18407879	RM21	19173094	Li et al (2005)
LOC_Os11g45180	U18	NBS-LRR disease resistance protein	Deep root ratio	11	PC41M14	17353847	PC48M15	18179510	Kamoshita et al (2002b)
LOC_Os11g45180	U18	NBS-LRR disease resistance protein	Deep root weight	11	PC48M15	17353847	PC31M8	18179510	Kamoshita et al (2002b)
LOC_Os11g45180	U18	NBS-LRR disease resistance protein	Penetrated root length	11	PC74M2	19563844	RG103	20337612	Ali et al (2000)
LOC_Os11g45180	U18	NBS-LRR disease resistance protein	Deep root weight per tiller	11	C477	5460751	EM17_10	6686558	Kamoshita et al (2002a)

LOC_Os11g45180	U18	NBS-LRR disease resistance protein	Maximum root length	11	G4001	22816378	RM254	23764518	Xu et al (2004)
LOC_Os11g45180	U18	NBS-LRR disease resistance protein	Maximum root length	11	RM206	22014679	RG1109	23155291	Kamoshita et al (2002a)
LOC_Os11g45180	U18	NBS-LRR disease resistance protein	Root growth rate in depth	11	RM332	2840213	RM167	4073313	Yue et al (2006)
LOC_Os11g45180	U18	NBS-LRR disease resistance protein	Root dry weight	11	C794	3033385	RG118	4414504	Xu et al (2004)
LOC_Os11g45180	U18	NBS-LRR disease resistance protein	Root dry weight	11	C794	3033385	RG118	4414504	Xu et al (2004)
LOC_Os11g45180	U18	NBS-LRR disease resistance protein	Deep root ratio	11	RM287	16767319	RM229	18408009	Yue et al (2006)
LOC_Os11g45180	U18	NBS-LRR disease resistance protein	Maximum root length	11	RM287	16767319	RM229	18408009	Yue et al (2006)
LOC_Os11g45180	U18	NBS-LRR disease resistance protein	Root growth rate in depth	11	RM287	16767319	RM229	18408009	Yue et al (2006)
LOC_Os11g45180	U18	NBS-LRR disease resistance protein	Root growth rate in depth	11	RM287	16767319	RM229	18408009	Yue et al (2006)
LOC_Os11g45180	U18	NBS-LRR disease resistance protein	Deep root weight	11	G257	17353847	ME2_6	19173094	Kamoshita et al (2002a)
LOC_Os11g45180	U18	NBS-LRR disease resistance protein	Deep root weight	11	G257	17353847	ME2_6	19173094	Kamoshita et al (2002a)
LOC_Os11g45180	U18	NBS-LRR disease resistance protein	Deep root ratio	11	G257	17353847	ME2_6	19173094	Kamoshita et al (2002a)
LOC_Os11g45180	U18	NBS-LRR disease resistance protein	Root pulling force	11	G257	17353847	RM21	19173094	Zhang et al (2001a)
LOC_Os11g45180	U18	NBS-LRR disease resistance protein	Deep root weight	11	R642	2015713	RZ141	4078514	MacMillan et al (2006)
LOC_Os11g45180	U18	NBS-LRR disease resistance protein	Maximum root length	11	R642	2015713	RZ141	4078514	Price et al (1999)
LOC_Os11g45180	U18	NBS-LRR disease resistance protein	Deep root ratio	11	ME6_7	19563844	ME7_2	22014851	Kamoshita et al (2002a)
LOC_Os11g45180	U18	NBS-LRR disease resistance protein	Deep root weight per tiller	11	ME7_2	19563844	EM18_19	22014851	Kamoshita et al (2002a)
LOC_Os11g45180	U18	NBS-LRR disease resistance protein	Root thickness	11	ME7_2	19563844	EM18_19	22014851	Kamoshita et al (2002a)
LOC_Os11g45180	U18	NBS-LRR disease resistance protein	Deep root ratio	11	RM286	383711	RM332	2840362	Yue et al (2006)
LOC_Os11g45180	U18	NBS-LRR disease resistance protein	Deep root ratio	11	RM286	383711	RM332	2840362	Yue et al (2006)
LOC_Os11g45180	U18	NBS-LRR disease resistance protein	Maximum root length	11	RM286	383711	RM332	2840362	Yue et al (2006)
LOC_Os11g45180	U18	NBS-LRR disease resistance protein	Root thickness	11	RM229	18407879	C189	21426217	MacMillan et

									al (2006)
LOC_Os11g45180	U18	NBS-LRR disease resistance protein	Root thickness	11	RM229	18407879	C189	21426217	Price et al (2002c)
LOC_Os11g45180	U18	NBS-LRR disease resistance protein	Root growth rate in volume	11	RM202	9001608	RM287	16767617	Yue et al (2006)
LOC_Os11g45180	U18	NBS-LRR disease resistance protein	Root growth rate in volume	11	RM202	9001608	RM287	16767617	Yue et al (2006)
LOC_Os11g45180	U18	NBS-LRR disease resistance protein	Root growth rate in volume	11	RM202	9001608	RM287	16767617	Yue et al (2006)
LOC_Os11g45180	U18	NBS-LRR disease resistance protein	Root growth rate in volume	11	RM202	9001608	RM287	16767617	Yue et al (2006)
LOC_Os11g45180	U18	NBS-LRR disease resistance protein	Root dry weight	11	RM202	9001608	RM21	19173094	Horii et al (2006)
LOC_Os11g45180	U18	NBS-LRR disease resistance protein	Root branching index	11	RM202	9001608	RM21	19173094	Horii et al (2006)
LOC_Os11g45180	U18	NBS-LRR disease resistance protein	Root thickness	11	OSR1	201993	RM202	9001898	Li et al (2005)

Supplementary Table 3. Primer sequences used in the study

**Primer Name**	**Primer Sequence (5’-3’)**
ARD4F	AGTAATCCATCCTCCTCATC
ARD4R	CTACTATCCGGCTATCCTAC
ARD4F for qRT-PCR	AGTAATCCATCCTCCTCATC
ARD4R for qRT-PCR	GGTACTGCTTGGAGGGTAGC
Hyg -F	TACACAGCCATCGGTCCA
CaMV35S-R	ACCTCCTCGGATTCCATTGC
Ubiquitin -F	AGAAGCGCAAGAAGAAGACG
Ubiquitin -R	GCGTCGTCCACCTTGTAGA
